# Comparative Whole-Genome Analysis of Clinical Isolates Reveals Characteristic Architecture of *Mycobacterium tuberculosis* Pangenome

**DOI:** 10.1371/journal.pone.0122979

**Published:** 2015-04-08

**Authors:** Vinita Periwal, Ashok Patowary, Shamsudheen Karuthedath Vellarikkal, Anju Gupta, Meghna Singh, Ashish Mittal, Shamini Jeyapaul, Rajendra Kumar Chauhan, Ajay Vir Singh, Pravin Kumar Singh, Parul Garg, Viswa Mohan Katoch, Kiran Katoch, Devendra Singh Chauhan, Sridhar Sivasubbu, Vinod Scaria

**Affiliations:** 1 GN Ramachandran Knowledge Center for Genome Informatics, CSIR Institute of Genomics and Integrative Biology (CSIR-IGIB), Mall Road, Delhi—110007, India; 2 Genomics and Molecular Medicine, CSIR Institute of Genomics and Integrative Biology (CSIR-IGIB), Mall Road, Delhi—110007, India; 3 National JALMA Institute of Leprosy and other Mycobacterial Diseases, Post Box No.101,Tajganj, Agra-282001, India; 4 Open Source Drug Discovery Unit, Council of Scientific and Industrial Research (CSIR), Anusandhan Bhavan, 2 Rafi Marg, New Delhi 110001, India; 5 Academy of Scientific & Innovative Research (AcSIR), 2, Rafi Marg, Anusandhan Bhawan, New Delhi 110001, India; National Institute of Infectious Diseases, JAPAN

## Abstract

The tubercle complex consists of closely related mycobacterium species which appear to be variants of a single species. Comparative genome analysis of different strains could provide useful clues and insights into the genetic diversity of the species. We integrated genome assemblies of 96 strains from *Mycobacterium tuberculosis* complex (MTBC), which included 8 Indian clinical isolates sequenced and assembled in this study, to understand its pangenome architecture. We predicted genes for all the 96 strains and clustered their respective CDSs into homologous gene clusters (HGCs) to reveal a hard-core, soft-core and accessory genome component of MTBC. The hard-core (HGCs shared amongst 100% of the strains) was comprised of 2,066 gene clusters whereas the soft-core (HGCs shared amongst at least 95% of the strains) comprised of 3,374 gene clusters. The change in the core and accessory genome components when observed as a function of their size revealed that MTBC has an open pangenome. We identified 74 HGCs that were absent from reference strains H37Rv and H37Ra but were present in most of clinical isolates. We report PCR validation on 9 candidate genes depicting 7 genes completely absent from H37Rv and H37Ra whereas 2 genes shared partial homology with them accounting to probable insertion and deletion events. The pangenome approach is a promising tool for studying strain specific genetic differences occurring within species. We also suggest that since selecting appropriate target genes for typing purposes requires the expected target gene be present in all isolates being typed, therefore estimating the core-component of the species becomes a subject of prime importance.

## Introduction

*Mycobacterium tuberculosis* complex (MTBC) is the etiologic agent of tuberculosis (TB), one of the largest killer infectious disease causing worldwide morbidity and mortality. Bacterial strains belonging to the same species vary considerably in their genetic repertoire. The complex encompasses a group of closely related gram-positive bacteria including, *M*. *tuberculosis* (Mtb) and *M*. *africanum*—the typical human pathogens [1,2]; *M*. *bovis*—the bovine form [3]; *M*. *canettii*—whose actual host is unknown; and several other lineages infecting mammals such as *M*. *microti*, *M*. *caprae*, *M*. *pinnipedii* [4] and *M*. *orygis* [5]. The species of the tubercle complex are not merely closely related but appears to be variants of a single species. TB manifests in a variety of clinically distinct and phenotypically diverse infections in humans with a distinctly long sub-clinical latency period [6,7]. Despite numerous improved diagnostic methods, and an effective combination therapy, the global emergence and spread of tuberculosis continues unabated, with an estimated 8.7 million people getting new infections every year [8]. This scenario has been complicated by widespread emergence of multidrug resistant (MDR) (resistance to first-line of Mtb drugs, isoniazid and rifampicin), extensively-drug resistant (XDR) (resistance to any member of the quinolone family and one of the second-line injectable drugs in addition to first-line drugs isoniazid and rifampicin) [9] and lately, totally drug resistant (TDR) (resistance to a wide range of TB drugs) strains [10,11]. The infection also forms a lethal combination when associated with clinical immunosuppressive states, which are either induced due to long-term treatment using immune-suppressants [12] or its synergy with Human Immunodeficiency Virus [13]. Recent studies on global phylo-geography of the pathogen and analysis of its virulence and drug resistance have suggested strain-specific genetic differences and a geographically constrained clonal population structure of MTBC [14,15]. Detailed studies of spoligotypes and associations with drug resistance phenotypes have been extensively reported in geographically limited isolates [16–18]. MTBC is also known to have a large arsenal of genomic features relating to the virulence, including its ability to persist and replicate in extremely hostile intracellular conditions and establish long-term infections in the host [7,19].

The availability of the complete genome sequence of Mtb laboratory strain H37Rv in 1998 [2], provided useful insights into the biology of the pathogen. The reference H37Rv genome, encodes for a repertoire of close to 4,000 protein-coding genes [2]. Previous comparative genome analysis of different strains has shown that such an approach could provide useful clues and insights into the genetic diversity of the species [20]. The availability of high throughput and cost effective nucleic acid sequencing technologies has opened up new possibilities towards understanding genomic diversity spanning a large number of members of the same species and has revolutionized the in-depth analyses of complex microbial populations [21–23]. Previous studies from other bacterial genera highlighted significant differences arising in the genomes of laboratory strains and clinical isolates [24]. Recent studies involving re-sequencing of a large number of clinical isolates have also provided key insights into the genomic variability of the pathogen. The study revealed that the variability among MTBC strains is more widespread than was initially anticipated [25]. Nevertheless, extensive characterization of the genomic structure and diversity in terms of gene repertoires of laboratory and clinical isolates has not been studied in detail. The recent availability of complete and near-complete sequences of clinical isolates of MTBC provides an interesting and novel opportunity to address the question of conservation and variation to understand the pangenome of the species. A pangenome is a union of entire genetic pool of several strains of a species under comparison, essentially consisting of a core genome containing genes and sequences shared in all strains and an accessory genome comprising of genes and sequences, which may be absent from one or more strains and genes that are unique to each strain [26].

In the present study, we elucidate the pangenome structure of *M*. *tuberculosis* complex using 96 strains, which comprise both complete as well as draft genomes including eight genomes of clinical isolates from India sequenced and assembled as part of this study. The data pool had MDR and XDR strains as well. We predicted genes for all the 96 strains and clustered their respective CDSs to reveal a hard-core, soft-core and accessory genome component of MTBC. Additionally, we also identified a subset of 74 gene clusters from the accessory component of MTBC, which was present in at-least a third of clinical isolates (i.e. >1/3^rd^ of total dataset), but absent from the laboratory maintained reference strains (Mtb H37Ra and H37Rv). The study demonstrates the power of genomics based approach enabling close assessment and detection of conserved gene sets within closely related strains of MTBC.

## Results

We analyzed a total of 96 strains in the present study to describe the pangenome of MTBC. Briefly, the data pool comprised of 25 complete reference genomes and 71 draft assemblies. The draft assemblies comprised of eight Indian strains derived from the ‘Open Source Drug Discovery (OSDD) open access repository’ and were sequenced and assembled in this study (Table 1). The sequencing and assembly protocols of the eight OSDD isolates has been described in detail in methods section. The 88 strains derived from public domain encompassed diverse geographical locations around the globe and were sequenced and assembled using different sequencing techniques and methodologies. Reference guided genome assemblies were excluded from our analysis as estimating the size of bacterial pangenome requires *de-novo* assemblies of the genome in complete or partial genomes with large contigs. A detailed account of the dataset including full organism name, their status of completeness, sequencing platform and coverage is presented in S1 Table.

**Table 1:** Salient features of the 96 *M*. *tuberculosis* complex genomes used in the present study.

SNo	Strain ID	Accession No.	Scaffolds	Predicted genes
1.	M. africanum GM041182	NC_015758.1	1	4041
2.	M. bovis AF2122/97	NC_002945	1	4009
3.	M. bovis AN5	AWPL01	70	4051
4.	M. bovis BCG str. ATCC 35733	AEZF01	32	4024
5.	M. bovis BCG str. ATCC 35740	AEZG01	36	4047
6.	M. bovis BCG str. ATCC 35743	AEZH01	28	4036
7.	M. bovis BCG str. China	AEZE01	29	4039
8.	M. bovis BCG str. Frappier	AKYQ01	178	4015
9.	M. bovis BCG str. Glaxo	AKYR01	327	4050
10.	M. bovis BCG str. Korea 1168P	NC_020245	1	4028
11.	M. bovis BCG str. Moreau	AKYS01	540	4505
12.	M. bovis BCG str. Pasteur 1173P2	NC_008769	1	4026
13.	M. bovis BCG str. Phipps	AKYT01	810	4103
14.	M. bovis BCG str. Prague	AKYU01	896	4114
15.	M. bovis BCG str. Sweden	AKYV01	1092	4147
16.	M. bovis BCG str. Tokyo 172	NC_012207	1	4034
17.	M. canettii CIPT 140010059	NC_015848	1	4070
18.	M. canettii CIPT 140060008	NC_019950	1	4059
19.	M. canettii CIPT 140070002	CAOL01	587	4105
20.	M. canettii CIPT 140070005	CAOM01	516	4058
21.	M. canettii CIPT 140070007	CAOO01	502	4208
22.	M. canettii CIPT 140070008	NC_019965	1	4062
23.	M. canettii CIPT 140070010	NC_019951	1	4124
24.	M. canettii CIPT 140070013	CAON01	542	4124
25.	M. canettii CIPT 140070017	NC_019952	1	4116
26.	M. orygis 112400015	APKD01	108	4017
27.	M. tuberculosis OSDD472	SRR786667	432	4108
28.	M. tuberculosis OSDD326	SRR786668*	450	4125
29.	M. tuberculosis OSDD630	SRR786188	411	4123
30.	M. tuberculosis OSDD386	SRR784917*	565	4229
31.	M. tuberculosis OSDD487	SRR786669*	383	4173
32.	M. tuberculosis 02_1987	ABLM01	22	4352
33.	M. tuberculosis 210	ADAB01	130	4231
34.	M. tuberculosis 43–16836	ATNF01	154	4149
35.	M. tuberculosis 7199–99	NC_020089	1	4075
36.	M. tuberculosis 94_M4241A	ABLL01	10	4302
37.	M. tuberculosis '98-R604 INH-RIF-EM'	ABVM01	16	4183
38.	M. tuberculosis BTB05-552	AEGC01	1	4109
39.	M. tuberculosis BTB05-559	AEGD01	1	4081
40.	M. tuberculosis C	AAKR01	4	4276
41.	M. tuberculosis CCDC5079.1	NC_017523	1	4190
42.	M. tuberculosis CCDC5079.2	NC_021251	1	4088
43.	M. tuberculosis CCDC5180	NC_017522*	1	4105
44.	M. tuberculosis CDC1551	NC_002755	1	4080
45.	M. tuberculosis CDC1551A	AELF01	53	4138
46.	M. tuberculosis CPHL_A	ACHP01	6	4195
47.	M. tuberculosis CTRI-2	NC_017524	1	4073
48.	M. tuberculosis CTRI-4	AIIE01~	44	4048
49.	M. tuberculosis EAI/OSDD271	AQQC01*	182	4248
50.	M. tuberculosis EAS054	ABOV01	6	4240
51.	M. tuberculosis F11	NC_009565	1	4085
52.	M. tuberculosis FJ05194	ANBL01~	112	4113
53.	M. tuberculosis GM 1503	ABQG01	17	4367
54.	M. tuberculosis GuangZ0019	ANFI01~	97	4128
55.	M. tuberculosis H37Ra	AAYK01	272	4292
56.	M. tuberculosis H37Ra	NC_009525	1	4095
57.	M. tuberculosis H37Rv	NC_000962	1	4086
58.	M. tuberculosis H37Rv	NC_018143	1	4077
59.	M. tuberculosis H37RvCO	AJSF01	1	4088
60.	M. tuberculosis K85	ACHQ01	1	4159
61.	M. tuberculosis KZN 1435	NC_012943*	1	4079
62.	M. tuberculosis KZN 4207	NC_016768	1	4065
63.	M. tuberculosis KZN 605	NC_018078~	1	4079
64.	M. tuberculosis NA-A0008	ALYG01	280	4375
65.	M. tuberculosis NA-A0009	ALYH01	310	4404
66.	M. tuberculosis NCGM2209	BADQ01	107	4195
67.	M. tuberculosis OSDD071	AHHX01/SRR786373*	393	4145
68.	M. tuberculosis OSDD105	AUXD02*	181	4448
69.	M. tuberculosis OSDD493	AVQJ01~	193	4303
70.	M. tuberculosis OSDD504	AHHY01/SRR786397*	631	4202
71.	M. tuberculosis OSDD518	AHHZ01/SRR786670	404	4132
72.	M. tuberculosis PR05	AOMG02	225	4142
73.	M. tuberculosis S96-129	AEGB01	1	4112
74.	M. tuberculosis SP21	AOUF01	30	4364
75.	M. tuberculosis str. Erdman = ATCC 35801	NC_020559	1	4095
76.	M. tuberculosis str. Haarlem	NC_022350	1	4063
77.	M. tuberculosis str. OSDD515	AUXC01*	127	4197
78.	M. tuberculosis SUMu001	ADHQ01	79	4188
79.	M. tuberculosis SUMu002	ADHR01	40	4246
80.	M. tuberculosis SUMu003	ADHS01	63	4175
81.	M. tuberculosis SUMu004	ADHT01	64	4178
82.	M. tuberculosis SUMu005	ADHU01	55	4170
83.	M. tuberculosis SUMu006	ADHV01	58	4193
84.	M. tuberculosis SUMu007	ADHW01	41	4160
85.	M. tuberculosis SUMu008	ADHX01	41	4183
86.	M. tuberculosis SUMu009	ADHY01	46	4153
87.	M. tuberculosis SUMu010	ADHZ01	52	4191
88.	M. tuberculosis SUMu011	ADIA01	56	4248
89.	M. tuberculosis SUMu012	ADIB01	93	4306
90.	M. tuberculosis T17	ABQH01	22	4543
91.	M. tuberculosis T46	ACHO01	8	4302
92.	M. tuberculosis T85	ABOW01	17	4421
93.	M. tuberculosis T92	ABLN01	31	4524
94.	M. tuberculosis UM 1072388579	AMXW01~	89	4035
95.	M. tuberculosis UT205	NC_016934.1	1	4120
96.	M. tuberculosis W-148	ACSX01	1	4270

*—Denotes MDR strains,

~—Denotes XDR strains.

### Strain characterization

The 96 MTBC strains comprised of five species—*M*. *tuberculosis*, *M*. *bovis*, *M*. *canettii*, *M*. *orygis* and *M*. *africanum*. The strains Mtb ATCC H37Rv and Mtb ATCC H37Ra maintained at ATCC (American Type Culture Collection) have been referred to as reference and/or laboratory strains in this study and any comparison attributing to H37Rv and H37Ra reference strains in our study is in context with these ATCC strains only. Total genome sizes of all strains ranged from 4.08MB to 4.57MB with an average GC content of 65.4%. Ten strains used in the present study were phenotypically characterized as Multi-Drug Resistant (MDR) out of which five were already described as MDR (Mtb CCDC5180, Mtb EAI OSDD271, Mtb KZN1435, Mtb OSDD105, and Mtb OSDD515) and the other five (Mtb OSDD326, Mtb OSDD 386, Mtb OSDD071, Mtb OSDD504 and Mtb OSDD487) are newly sequenced strains on which Drug-Susceptibility Testing (DST) was done using a panel of 12 Mtb drugs comprising of first-line, second-line and combination drugs. Additionally, six strains were characterized as Extensively-Drug resistant (XDR), Mtb CTRI-4, Mtb FJ05194, Mtb GuangZ0019, Mtb KZN605, Mtb OSDD493 and Mtb UM1072388579. The detailed results of DST profiling are presented in S1 Fig. The spoligotyping results on the 8 Indian strains suggested that three of them belonged to the clade CAS1_Delhi, which forms a major class of Mtb clinical isolates from Northern India, while two had novel spoligotype patterns (S1 Fig).

### Genome sequencing and assembly

Estimating the size of the bacterial pangenome requires *de-novo* assemblies of the genome into complete or partial genomes with large contigs. *De-novo* genome assembly also provides an opportunity to reveal differences in newly sequenced strains with respect to reference genomes. A recent report [27] suggests that the majority of genes in the prokaryotic genomes can be easily re-constructed with short read sequencing technologies. The 96 MTBC genome sequences (Table 1) used in this study comprised of 25 complete reference strains sequenced and assembled elsewhere. The reference genomes included the two laboratory strains Mtb H37Ra and Mtb H37Rv. The 71 draft genome assemblies obtained from NCBI comprised of scaffolds ranging from 4–1092. The remaining 8 strains sequenced in this study are part of the Open Source Drug Discovery Open Access Repository and correspond to IDs: Mtb OSDD472, Mtb OSDD326, Mtb OSDD071, Mtb OSDD504, Mtb OSDD630, Mtb OSDD386, Mtb OSDD487 and Mtb OSDD518. DNA library preparation and sequencing was performed using standard protocols as detailed in methods section. Sequencing reads from the eight newly sequenced strains have been deposited at the Sequence Read Archive (SRA) service of NCBI with accessions SRR784917, SRR786188, SRR786373, SRR786397, SRR786667, SRR786668, SRR786669 and SRR786670.

The 101bp paired-end raw sequence reads were *de novo* assembled at different k-mers as detailed in materials and methods. Majority of the samples had the best assembly at k-mer value of greater than 50 with the highest N50 values ranging from 14Kbps-28Kbps. The entire assembly resulted in draft genomes of over 4.3Mbps. The genome assembly statistics for the strains are summarized in Table 2. The finest contig assemblies generated from the *de novo* assembly are available at http://genome.igib.res.in/Mtb_Pangenome.

**Table 2:** Genome Assembly Statistics of the eight Indian strains sequenced and assembled as part of the present study.

Strain_id	Raw_reads	k-mer	N50 size (bp)	Total contigs (bp)	Largest contig size (bp)	Assembly size (bp)
Mtb OSDD472	34645616	63	28444	432	98618	4360369
Mtb OSDD326	22694588	59	26414	450	94773	4343218
Mtb OSDD071	19113318	55	25700	393	103182	4324807
Mtb OSDD504	12027546	51	14067	631	49228	4291530
Mtb OSDD630	11407172	53	25990	411	91835	4300760
Mtb OSDD386	9535006	41	15827	565	72092	4339775
Mtb OSDD487	9234034	53	26861	383	84901	4356171
Mtb OSDD518	9495612	55	25234	404	105956	4316163

### Pangenome estimation and analysis

#### Gene prediction and clustering

Whole genome sequences (WGS) of the complete MTBC genomes and scaffold/contig assemblies of draft genomes were used to predict their coding sequences (CDS) / Open Reading Frames (ORFs). Standard bacteria/archaea translation codes were used for CDS prediction. An account of the number of CDS predicted along for each of the 96 strains is provided in Table 1. The predicted gene pool of the 96 strains was used to estimate the pangenome of Mtb. The gene repertoire of each of the 96 Mtb isolates comprises of 4,162 ± 119 genes (mean ± standard deviation). For some genomes the number of predicted genes could be an over-estimation because of the draft nature of the genomes and lower quality scaffolds with stretches of N’s. The protein translations of CDSs predicted from the complete genomes and scaffold assemblies of draft genomes were clustered based on sequence identity using CD-HIT as detailed in the methods section. The clustering approach resulted in a total number of 8,099 Homolog Gene Clusters (HGCs), which represents the MTBC “pangenome”. The clusters are referred to as HGCs because a given cluster can have paralog sequences as well.

#### Hard-core versus Soft-core

Genes shared amongst all strains of a species under study is referred to as the ‘hard-core’ genome. A ‘soft-core’ genome of a species is the number of genes shared amongst a large proportion of genomes under study, say >90% of the strains under consideration. The soft-core could perhaps serve to be more biologically relevant in view of the large number of draft quality genomes used in the analysis. The number of HGCs shared amongst all 96 MTBC strains i.e. the ‘hard-core’ comprised of 2,066 HGCs and remaining 6,033 HGCs formed the accessory gene pool. In order to choose an optimum threshold for defining the ‘soft-core’, we checked out for the presence of essential genes in the core component. Genes which are critical for the growth and survival of an organism are considered as essential genes for that organism. Three independent studies differing in the mycobacterium growth conditions have been performed in the past to define the essential genes of *Mycobacterium tuberculosis* H37Rv [28–30] and have been catalogued in the Database of Essential Genes (DEG) [31].

We performed a BLAST search of all 8,099 representative cluster sequences against the essential genes of Mtb in the DEG database and identified 2,149 matches. Since the BLAST search retrieved multiple and redundant matches we selected only the ones present in all the three studies. There were 348 overlapping genes from the three studies and 597 sequences from our data had homology with these 348 genes. An optimal ‘soft-core’ was defined at 95% where maximum number of our essential gene matches was falling into the core component (S2 Fig Thus in our study, the ‘soft-core’ component of MTBC is defined as clusters present in at least 95% of the strains. The soft-core of MTBC comprises of 3,374 HGCs and 4,725 HGCs are present in the accessory component.

#### The global phylogeny of MTBC

**Fig 1:**
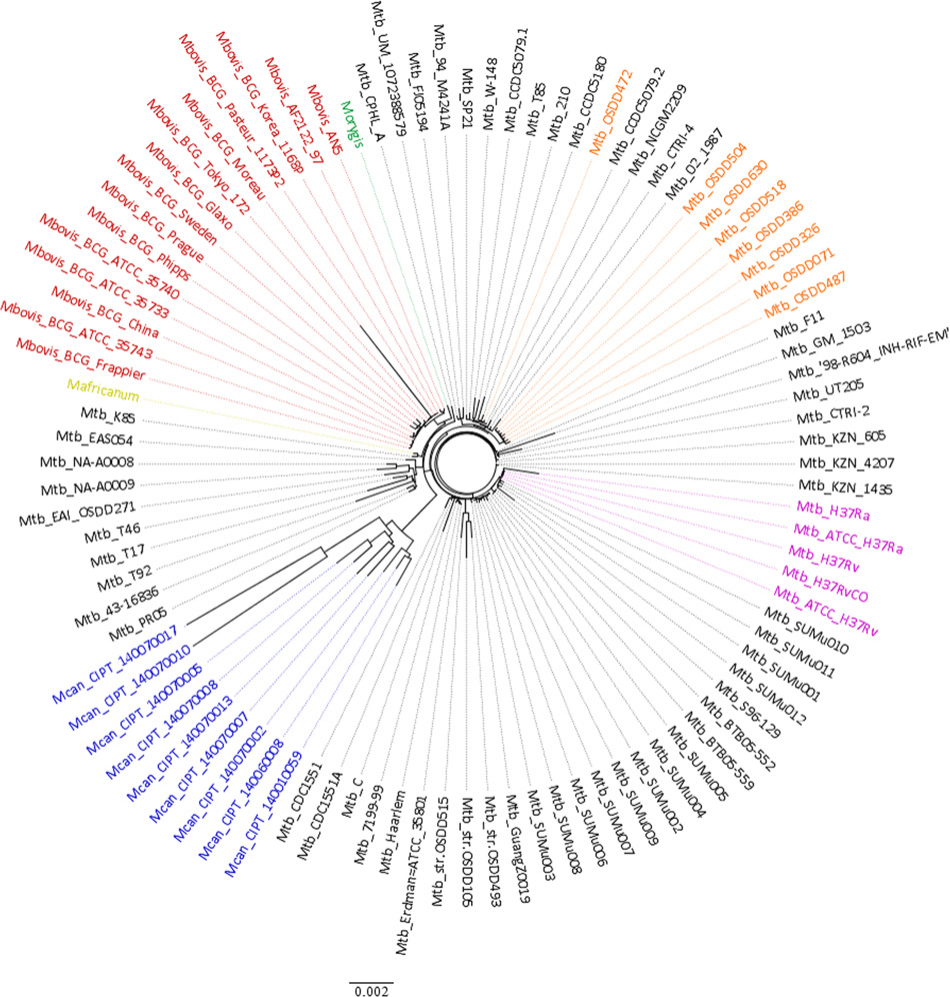
Core-gene Tree. The un-rooted MTBC tree was created from alignment of 971 orthologous core-genes from 96 strains. A tree with Bootstrapped values is presented in S3 Fig (details in text).

We investigated the genetic diversity within MTBC strains by inferring phylogeny based on genes. In contrast to the single gene based trees which have low inter-species discriminatory power, multi-gene approaches offer more robust phylogenetic trees [32,33]. In order to infer phylogeny of MTBC strains, we used the hard-core component i.e. genes shared amongst all the isolates under consideration. From the 2,066 hard-core, we removed the HGCs having paralogs thus leaving 971 orthologous gene clusters (OGCs) in the hard-core. CDS translations of the 971 OGCs from all 96 strains were subjected to multiple sequence alignment followed by bootstrapping, generating a total of 100 resamples. Pairwise differences amongst sequences were estimated and the resultant distance matrix was used to build the tree. The original and the resampled trees were compared and the resultant tree referred here as the core-gene tree, is presented in Fig 1 and the tree with comparative bootstrapped values is presented in S3 Fig.

The resultant tree of different species of MTBC is in concordance with the previously published reports where *M*. *canettii* formed the most ancient lineage of the MTBC [4] and in our results also, all strains of *M*. *canettii* formed a distinct clade. *M*. *africanum* was found to be more closely related to animal adapted strains of MTBC depicting similar evolutionary history as of *M*. *bovis* and *M*. *orygis* which has been reported earlier as well [34]. Except Mtb OSDD472, all other Indian clinical isolates sequenced in this study (orange colored in Fig 1) were all found to be clustering in a common clade and appeared to be very closely related. The Mtb reference strains H37Rv and H37Ra also clustered distinctly, depicting their close association. We found that the phylogenomic relationship between human and animal adapted strains and species identified by using the core-gene component was largely consistent with previous reports [4,34,35] and additionally such a multi-gene approach served to better resolve strain specific genomic differences.

#### Core and accessory genome size evolution

**Fig 2:**
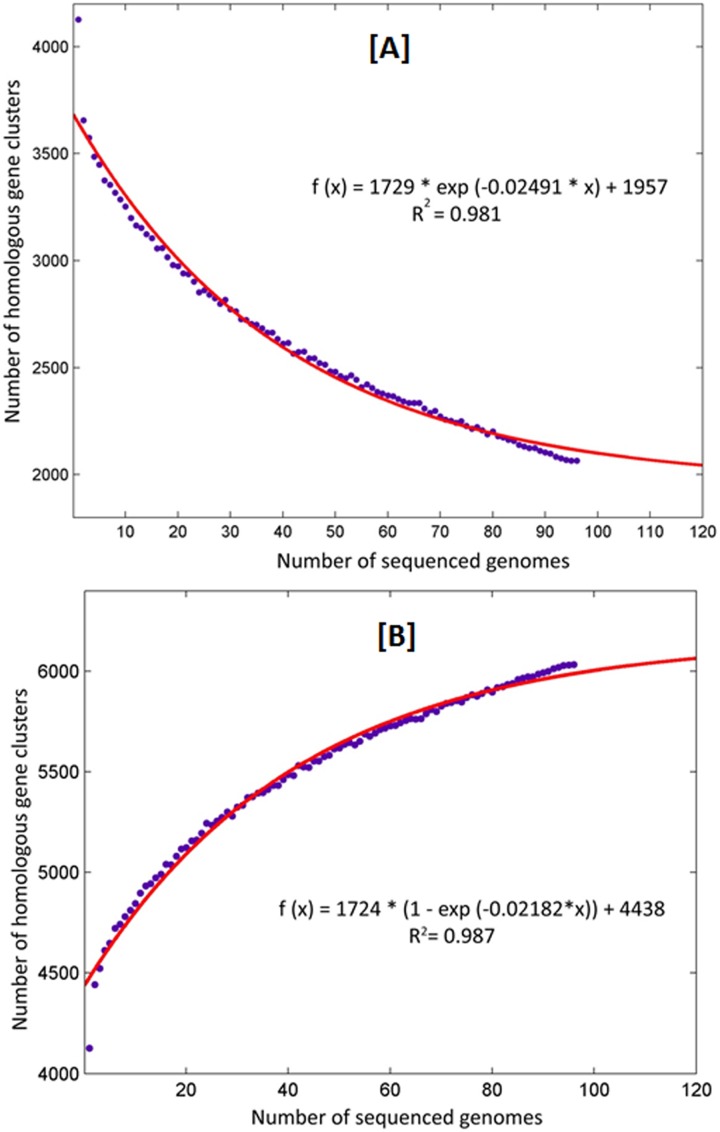
Core and accessory genome size evolution. (A) Each point indicates the number of HGCs conserved in a genome. The red line indicates an exponential decay function based on the median values of core HGCs when each time a new genome is added to the analysis. (B) Accessory genome of MTBC. The MTBC has an open pangenome model.

In view of defining the size of MTBC pangenome the primary question that arises is whether sufficient number of genomes has been sequenced to describe the core and accessory gene content of the species. For this we observed the change in the core and accessory gene component as a function of their size with increasing number of sampled genomes over the entire 96 genomes (Fig 2). The total genome component of the 96 MTBC strains was analysed to study the core and accessory genome size evolution in terms of exponential decay and growth models. The models are based on the median values of the conserved and accessory genome HGCs which in turn are obtained from the random permutations of genome comparisons and limiting the number of possible combinations to 100, for each new genome being added. The exponential decay model in Fig 2A suggests that the number of core HGCs tends to approach a plateau near 2,000 HGCs whereas the accessory HGCs tends to reach a plateau near 6,000 HGCs (Fig 2B) for the 96 strains under comparison. Since there is no distinctly sharp plateau formation, we estimate that the MTBC has an open pangenome i.e. the number of distinct genes found in MTBC strains is infinite as opposed to finite number of genes in a closed pangenome.

#### Annotation distribution of pangenome of MTBC

**Fig 3:**
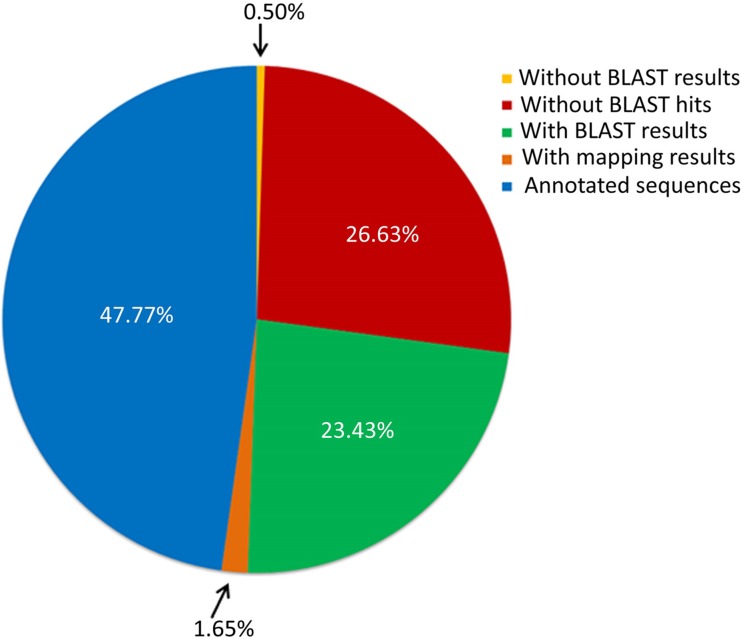
Summary of sequence annotation statistics from BLAST2GO. Representative sequences from all the 8,099 HGCs were subjected to annotation out of which 47.77% (3,869) sequences were annotated with GO slim terms, 26.63% (2,157) sequences were without any BLAST hits, 23.43% (1,898) sequences had only blast results but didn’t had annotation and 1.65% (134) sequences retrieved mapping results but were without GO slim terms. A small fraction of 0.5% (41) sequences failed to fetch BLAST results.

Each of the HGCs has a representative sequence which is the parent sequence of every cluster. All HGCs (i.e. 8,099) were annotated by querying their corresponding representative sequences against BLAST2GO as described in methods section. The overall annotation distribution (Fig 3) obtained from BLAST2GO showed that out of the 8,099 protein sequences, 47.77% (3,869) sequences were fully annotated with GO slim terms. 26.63% (2,157) sequences were without any BLAST hits (i.e. the sequence had absolutely no homology to any of the sequences present in the NCBI databases). Based on the results of BLAST hits obtained, the gene ontology mapping process retrieved GO terms distributed in BLAST matches. 23.43% (1,898) sequences failed to retrieve any GO terms associated with them.

#### Analysis of the soft-core component of the pangenome

In a total of 3,374 soft-core sequences, 2,921 sequences had GO terms associated with them (i.e. fully annotated) and the remaining ones had one or the other result missing (as in Fig 3 pie). The soft-core encompassed majority of the essential genes of Mtb as described above. The major COG (Clusters of Orthologous Groups) classes represented in the essential genes were E (amino acid transport and metabolism), J (translation, ribosomal structure and biogenesis), H (coenzyme transport and metabolism), R (general function), C (energy production and conversion), and M (cell wall or membrane or envelope biogenesis). A detailed account of the spread of the COG categories of essential genes is in S4 Fig.

**Fig 4:**
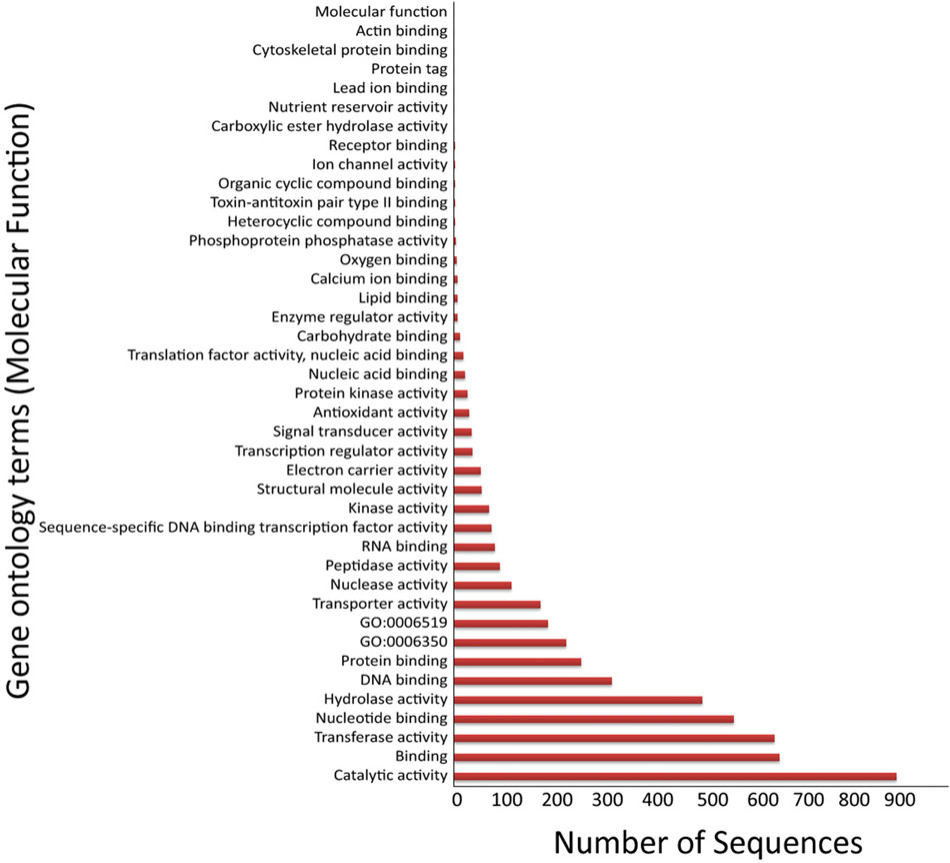
Molecular function GO annotations of the soft-core component. GO annotation for Biological process and Cellular component is provided in S5 and S6 Figs.

The three main Gene ontology terms under which all genes and gene products are represented include molecular function, cellular component and biological process. The 3,374 soft-core HGCs were annotated with a total of 14,297 GO terms out of which 93 were unique GO categories. Each sequence can have multiple GO terms associated with it, therefore representing redundant terms. A total of 5,023 sequences were spread over 41 GO terms of molecular function category and a distribution of these terms in the soft-core component is presented in Fig 4. The number of sequences in each GO term for the other two GO categories i.e. biological process and cellular component is represented in S5 and S6 Figs along with their annotation terms.

#### Analysis of the variable component of the pangenome

**Fig 5:**
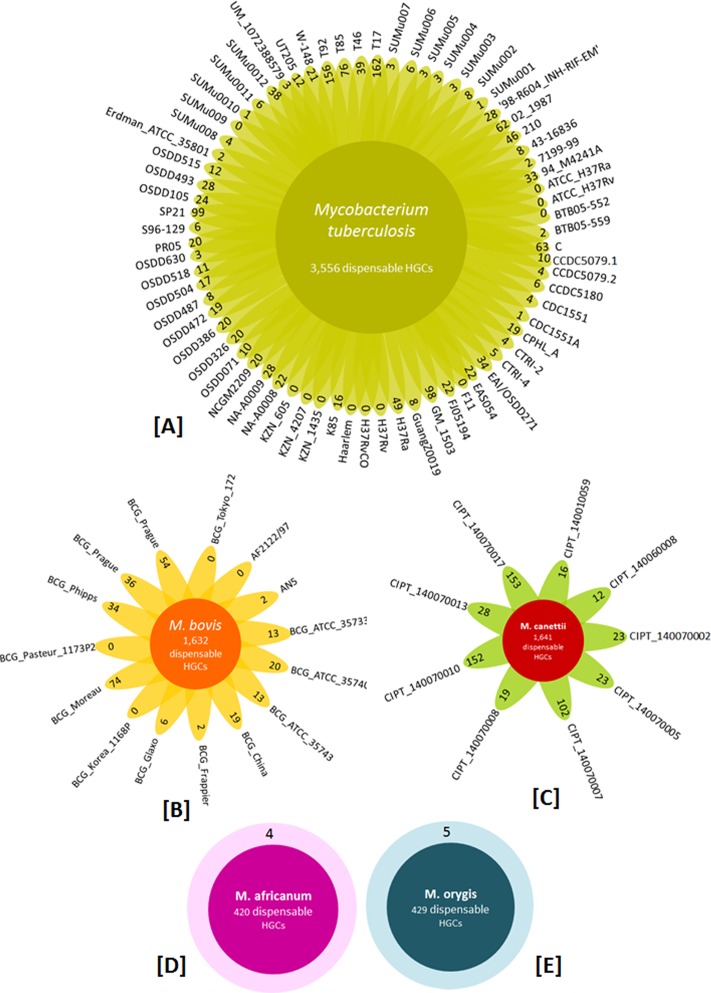
The accessory genome of MTBC. The flower plots depict the distribution of accessory genome HGCs across different species of MTBC. (A) Flower plot showing number of accessory HGCs present in Mtb (in center) and number of species-specific genes in the leaves. (B) Number of species-specific genes of *M*. *bovis* in leaves and total accessory HGCs in center. (C) *M*. *canettii* has accessory HGCs in center and species-specific genes in leaves. (D and E) The genomes of *M*. *africanum* and *M*. *orygis* have four and five species-specific genes respectively (outer circle) and total accessory HGCs in the center.

The soft-accessory genome of MTBC comprises of 4,725 HGCs. The 4,725 clusters were investigated to identify clusters shared among any given strain pair, unique to each strain and clusters with proteins present in greater than 1/3^rd^ of the clinical isolates but absent in the two laboratory strains i.e. Mtb H37Rv and Mtb H37Ra. A distribution of the accessory genome component in different species of MTBC is depicted in Fig 5. Single strain of *M*. *africanum* and *M*. *orygis* are present in 420 and 429 HGCs respectively, 70 isolates of *M*. *tuberculosis* are variably spread over 3,556 HGCs, 15 strains of *M*. *bovis* are present in 1,632 HGCs and 9 isolates of *M*. *canettii* are spread over 1,641 HGCs. The numbers in the center of flower plots and circular plots (Fig 5) are overlaps amongst species and are not unique to the species. The number of genes unique to a particular strain is represented in the leaves of the flower plots and in outer circles in case of *M*. *africanum* and *M*. *orygis*. It was found that most of the genomes with a status of ‘complete sequence’ had a very low number of unique genes as compared to draft quality genomes which had higher number of unique genes.

Though *M*. *canettii CIPT140070017* and *M*. *canettii CIPT140070010* have a complete genome status but they showed a high number of unique genes (153 and 152 respectively) in them. The possible reasons behind this could be presence of stretches of un-assigned bases denoted by N’s in the sequence or the bigger genome size of these two genomes (4.53 and 4.52MB) as compared to the average genome size of other *M*. *canettii* (4.38MB) genomes. We closely inspected the predicted functional annotation of these *M*. *canettii* unique genes obtained from BLAST matches. Majority of the BLAST hits were either self matches of the protein or they showed homology with proteins of other species of mycobacterium such as *M*. *kansasii*, *M*. *gastri*, *M*. *gilvum*, *M*. *marinum*, *M*. *yongonense*, *M*. avium, *M*. *abscessus*. Some of the proteins also showed homology with other bacterial species such as *Glaciibacter superstes*, *Gordonia kroppenstedtii*, and *Streptomyces griseus* etc.

A closer look into unique genes of all other genomes suggested that there could be multiple factors that can account for this intra- and inter- species variability such as genome size, nature of genomes (draft, complete or finished), size of contigs, genome scaffolds generated by introduction of N’s, sequence coverage, etc (S1 Table) and at present it is difficult to generalize or streamline the basis of this variability and also to differentiate true genes from artefacts.

**Fig 6:**
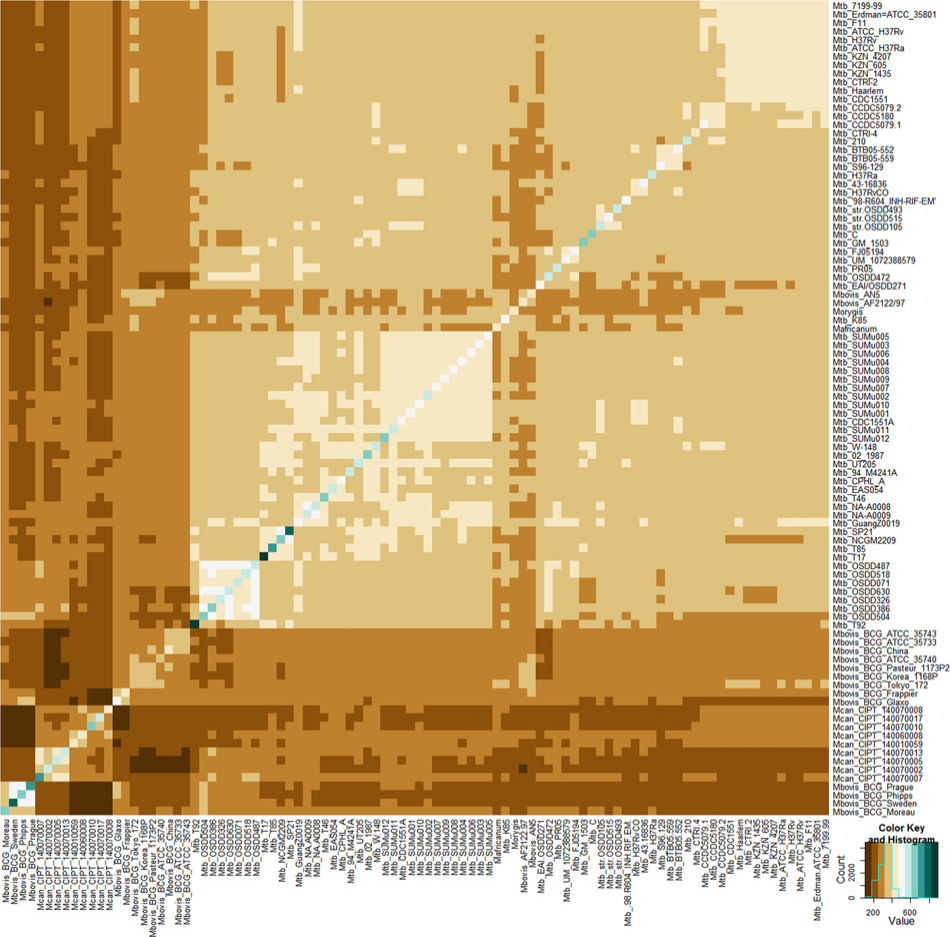
Overlap of accessory orthologous clusters shared within each strain pair. The diagonal represent the number of clusters present in any given strain and divides the data into exactly similar halves. Color key indicates the distribution of clusters. The sharing is irrespective of the cluster being present in any other strain. The minimum number of shared clusters is 109 clusters between Mcan CIPT 140070008 and Mbovis BCG Phipps and maximum (521) is between Mbovis BCG Sweden and Mbovis BCG Prague9.

An overlap of the number of accessory clusters shared within a strain pair is presented in Fig 6. The diagonal shows the total number of accessory clusters present in any strain being considered. The highest number of accessory clusters are present in the strain Mtb T17 (897) and the minimum number is present in Mbov BCG Korea (364). The upper and lower triangles are mirror images and show the number of overlapping clusters in each strain pair. The minimum number of overlapping clusters is 109 between Mcan CIPT 140070008 and Mbov BCG Phipps and maximum is 521, between Mbov BCG Sweden and Mbov BCG prague. The overlapping clusters formed a symmetrical matrix i.e. a square matrix which is equivalent to its transpose.

#### Clusters of genes absent in the H37Rv and H37Ra genome

**Fig 7:**
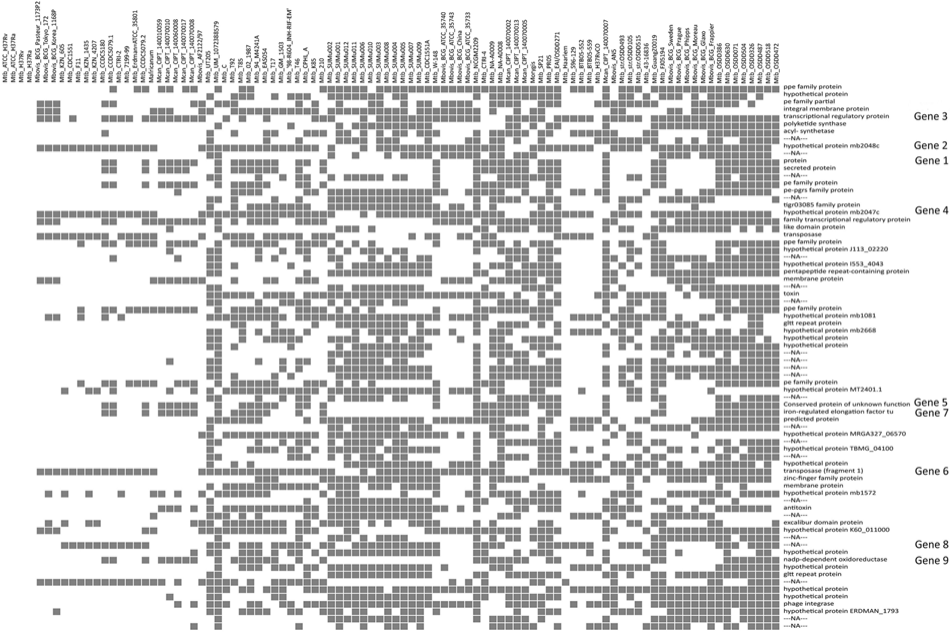
The heatmap shows 74 HGCs absent from reference strains Mtb ATCC H37Ra and Mtb ATCC H37Rv but present in most of the clinical isolates. Predicted annotation of each of the cluster family is also represented. The genes validated using PCR are also shown adjacent to the annotations.

We further analyzed the differences in the gene pool of clinical isolates (from across the globe) and laboratory maintained strains by looking out for genes that were conspicuously missing in the genome assemblies of the laboratory strains, H37Rv and H37Ra, but were present in at-least or greater than 1/3^rd^ of the clinical strains. We obtained 74 clusters meeting this criterion from the accessory genome of 4,725 HGCs. A cross-validation of the 74 clusters against whole genome sequences of H37Rv and H37Ra was done using BLAT [37]. We identified 10 clusters which had absolutely no match with H37Ra and H37Rv genomes, whereas the other 64 shared partial homology with H37Rv and H37Ra in variable regions of the genomes with a sequence length difference in the alignment. Fig 7 shows the 74 clusters absent from H37Ra and H37Rv (first 4 columns in Fig 7) but present in at-least 1/3^rd^ of the clinical isolates along with their annotations.

#### Experimental validation of the genes in the pangenome specific to clinical isolates

**Fig 8:**
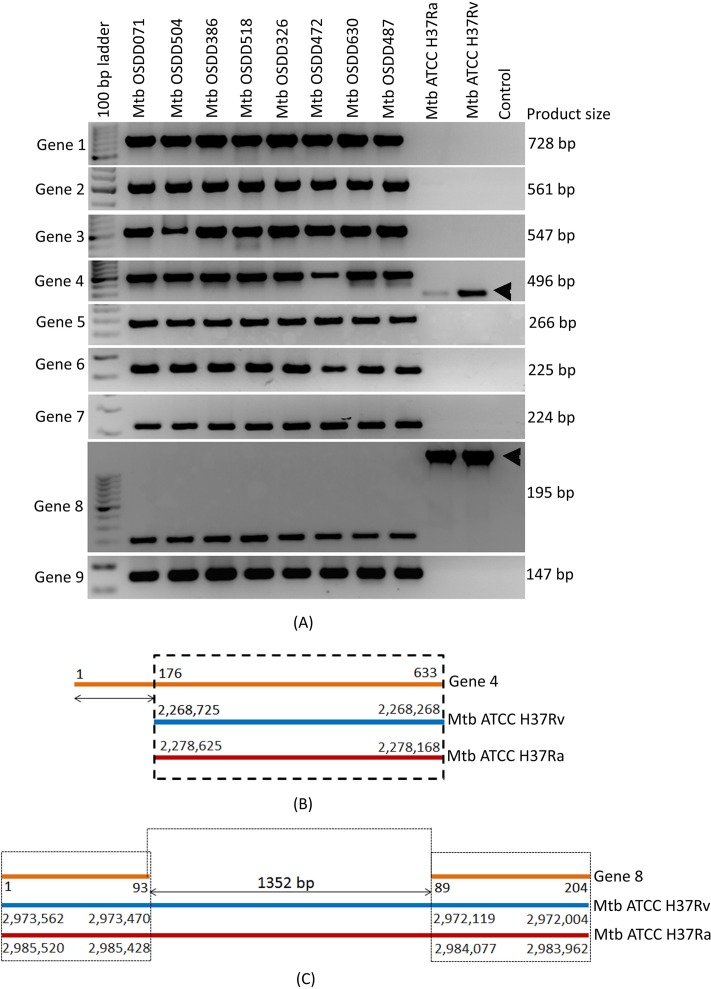
(A) PCR validation of the 10 candidate genes (subset of 74 clusters identified) in 8 Indian clinical isolates (OSDD strains) and reference strains ATCC H37Ra and ATCC H37Rv. Arrow heads indicate the variably sized products in ATCC H37Ra and ATCC H37Rv genomes. Gene 4 and Gene 8 shares partial homology with laboratory strains and showed the presence of a differently sized product in ATCC H37Rv and ATCC H37Ra. (B) The alignment of Gene 4 against reference genomes showed an insertion of 175bp sequence in Gene 4 of OSDD strains with respect to reference strains. (C) The alignment of Gene 8 against reference genomes showed a deletion of 1,352bp sequence in Gene 8 of OSDD strains.

We randomly picked 9 candidate HGCs sequences (Fig 7, genes 1–9) from the 74 clusters which were present in most of the clinical isolates and employed a classical PCR approach to experimentally validate these putative genic loci. PCR validation was performed on genomic DNA of the eight Indian OSDD strains sequenced in this study and laboratory strains Mtb ATCC H37Ra and Mtb ATCC H37Rv procured from ATCC. We designed primers for the 8 available OSDD strains using primer BLAST [37]. A detailed account of the forward and reverse primers used for the validation and their expected product sizes are summarized in S2 Table. All primers provided amplification of genomic DNA in expected sizes, substantiating and validating the presence of these genes in the strains under consideration. The PCR result for these 9 gene sequences are shown in Fig 8. Out of the 9 genes which were PCR validated, 7 genes were absolutely absent from H37Rv and H37Ra whereas 2 genes (Gene 4 and gene 8) displayed appearance of differentially sized gene products (these 2 genes shared partial homology with H37Ra and H37Rv).

The PCR amplification of Gene 4 showed presence of differentially sized products in Mtb ATCC H37Rv and Mtb ATCC H37Ra genomes (Fig 8A). In order to reveal the nature of these differentially sized products we used the predicted ORF sequence of Gene 4 (annotated as hypothetical protein) to align against the reference whole genomes of Mtb ATCC H37Rv and Mtb ATCC H37Ra using BLAST [38] (Fig 8B). The sequence alignment of Gene 4 (Texts C and D in S1 File) on the negative strand of Mtb ATCC H37Rv and Mtb ATCC H37Ra showed an insertion sequence of 175bp in Gene 4 of OSDD strains in comparison to the reference genomes. Detailed insights into the genomic loci of Gene 4 alignment with reference strains showed partial overlapping of Gene 4 with hypothetical proteins in Mtb ATCC H37Rv and Mtb ATCC H37Ra thereby resulting in the formation of differentially sized gene products (Figures A and B in S1 File). The PCR results of Gene 8 also showed differentially sized products in Mtb ATCC H37Rv and Mtb ATCC H37Ra genomes (Fig 8C). The alignment of the predicted ORF sequence of Gene 8 (no annotation) against the reference whole genomes of Mtb ATCC H37Rv and Mtb ATCC H37Ra (Texts C and D in S2 File) showed a deletion of 1,352bp sequence in Mtb OSDD strains. The genomic loci where Gene 8 aligns, majorly consists of repeat region and IS6110 transposase subunits in Mtb ATCC H37Rv and Mtb ATCC H37Ra (Figures A and B in S2 File). The corresponding insertion and deletion sequences of the putative ORFs of Gene 4 and Gene 8 respectively were confirmed by di-deoxy sequencing of their PCR products in Mtb OSDD472, Mtb ATCC H37Rv and Mtb ATCC H37Ra (Texts A and B in S1 and S2 Files).

The PCR results of Gene 4 and Gene 8 highlights the active role of genome re-arrangements taking place in the Mtb genomes and how the pathogen evolves by means of structural variations. Predicted functional annotation of the 9 candidate genes (Table 3) showed that ‘Gene 9’ is a NADP-dependent oxido-reductase. Gene 1, 2, 4 and 5 are hypothetical proteins whereas Gene 8 has no annotation associated with it. Genes 3 and 7 are involved in transcriptional and regulatory activity respectively and Gene 6 is a transposase involved in DNA binding.

**Table 3:** Summary of gene clusters absent from Mtb ATCC H37Ra and Mtb ATCC H37Rv.

Gene Id	Expected product size (bp)	Predicted function
Gene 1	728	Protein
Gene 2	561	Hypothetical protein
Gene 3	547	Transcriptional regulator
Gene 4	496	Hypothetical protein
Gene 5	266	Conserved protein
Gene 6	225	Transposase
Gene 7	224	Iron regulated Elongation factor tu
Gene 8	195	NA
Gene 9	147	NADP-dependent oxidoreductase

## Discussion

The close association of different species of the MTBC complex presents a challenging issue in understanding the genetic pool of the species. The difficulty is further enhanced by the fact that bacteria exchange genetic material in unique and unusual ways making the assessment of their core and accessory gene repertoire evolution even more complex. The study of genetic variability within natural population of pathogens may provide insight into their evolution and pathogenesis. Comparative genome analysis of the species in terms of variation in the distribution of SNPs, Indels, CRISPR-cas locus and nucleotide diversity has shown that their evolution is a complex process [35]. Also high level of sequence variation in the PE/PPE genes has showed absence of selective constraints and thereby suggesting neutral evolution [39]. The laboratory strain, *M*. *tuberculosis* H37Rv, has long been considered as the representative reference template for tuberculosis and has been extensively used to uncover potential drug targets [40,41]. However, selecting appropriate target gene/s for typing purposes requires that the expected target gene should be present in all isolates being typed. Therefore, estimating the core-component of the species becomes a subject of prime importance. Over the course of evolution genomes can undergo many large and small scale changes. For instance, recombination causes genome rearrangements, horizontal transfer introduces new sequences, and deletions remove segments of the genome [42,43]. Structural changes in the genome of closely related species have been reported in literature [44,45]. Such genome structural changes could also have phenotypic correlates [46]. Earlier studies have shown the emergence of significant genetic differences in genomes of laboratory strains and clinical isolates [24,25]. The newly emerging strains of a pathogen may have genomic differences, which cannot be addressed adequately using a sole reference genome. Over the past few years, WGS has widely been adopted for identifying genome-wide differences between related species [44,45], and significant work has been done for establishing WGS as a standard typing tool [47].

The current work not only complements previously done studies [35,39,48] but also provides an extensive characterization of the genomic structure of a large number of Mtb isolates from across the globe and highlights the rich diversity present in their genetic catalogue. Though relatively low sequencing costs have provided useful means to re-sequence genomes and simultaneously perform large scale comparative studies on pathogenic isolates [21,49] but the quality of the sequenced genomes remains a major concern. There is always a possibility of accidental incorporation of sequencing errors due to differences in sequencing protocols as well as genome assembly methods. Thus owing to the large number of draft quality genomes used in this work, we describe both hard-core and soft-core genome component of MTBC.

Understanding the differences existing within MTBC and evolutionary forces shaping these differences is not only crucial for uncovering its success as a pathogen but it is also expected to reveal key virulence mechanisms if any [50]. In the present study, we used a pangenome approach to identify a core set of genes which is shared by 96 strains of MTBC, and included most of the key essential genes of the bacterium. Our study comprised of diverse strains of MTBC from across the globe which included 10 MDR and 6 XDR strains and 8 Indian clinical isolates sequenced and assembled in this study. Global phylogeny based on a multi-gene approach involving 971 genes revealed the evolutionary relatedness of the 96 strains. The multi-gene tree was able to clearly differentiate between human- and animal- adapted strains and also phylo-geographically separated strains such as the newly sequenced 8 OSDD strains which formed a distinct clade and the laboratory strains clustering together showing their close association.

The MTBC pangenome comprise of 8,099 HGCs of which 2,066 gene clusters were present in the hard-core (100%) and 3,374 HGCs in the soft-core genome which was shared amongst 95% of the total strains analyzed. The variable component of the pangenome comprised of 4,725 gene clusters. Previous studies performed by Trost *et al* and Zakham *et al* on fourteen and twenty one mycobacterial genomes respectively have also shown that the mycobacteria has a fairly small core genome as compared to the accessory genome component [48,51]. From the accessory genome, a subset of 74 clusters was identified which was not represented in the reference laboratory templates, Mtb ATCC H37Rv and Mtb ATCC H37Ra, but were present in most of the clinical isolates being analyzed. We confirmed the complete absence of 7 candidate gene sequences from the laboratory templates using PCR and 2 genes sharing partial homology and having differentially sized gene products suggesting probable genome re-arrangement events of insertion and deletion. We argue that using a sole reference genome based approach could have severe limitations with under-representation of the actual repertoire of potential drug targets.

Our data suggest that a pangenome based approach applied to whole genome sequences can not only identify conserved genome regions but also detect rich information on genomic differences existing within strains of same species as has also been shown from previous comparative genomic studies on Mtb [35,48,51]. In a recent study by Liu *et al*, a comparative analysis of 7 newly sequenced clinical isolates and 7 complete reference genomes of Mtb showed that genomic variations might play an important role in the genomic plasticity of Mtb [35]. Such comparative genomics approaches are a promising tool for studying strain specific genetic differences occurring within species. With the availability of cheaper and high-throughput DNA sequencing techniques, there are a large number of MTBC strains being sequenced and raw sequencing reads being deposited in SRA [52]. Therefore, we hope that we will be able to extend these analyses encompassing a much larger dataset with more representative sequences corresponding to various geographical regions and strain types to elucidate the geographical signatures of pangenome components. We propose that a pangenome approach could identify novel and functionally relevant subsets of gene clusters with distinct functional characteristics and could provide a starting point towards re-looking at standard drug target discovery pipelines, which currently rely heavily on reference or template genomes.

## Materials and Methods

### The Strains and DNA isolation

A total of 96 strains from the Mtb complex were used in this study to construct and analyse the Pangenome architecture of MTBC. Out of these 96 genomes (data acquisition: 30-December-2013), 25 strains comprised of the whole genome sequences of MTBC and were obtained from the Refseq database of NCBI [53], 63 were draft genome assemblies present in the form of Scaffolds and/or contigs in NCBI whereas the remaining 8 strains were clinical isolates from India (Table 1). The DNA of the 8 strains was procured from the Open Source Drug Discovery Open Access Repository and corresponds to strain IDs OSDD472, OSDD326, OSDD071, OSDD504, OSDD630, OSDD386, OSDD487 and OSDD518. The strains were originally isolated at the National JALMA Institute of Leprosy and other Mycobacterial Diseases, Agra, India. Genomic DNA was isolated using the *Mycobacterium tuberculosis* genomic DNA isolation kit following manufacturer’s instruction (Premas Biotech, India, Cat No: PB-MTB-0200). Briefly, one ml of culture was centrifuged at 6,000 rpm for one min and supernatant was discarded. The pellet was re-suspended in 200 ul of WB1 buffer and incubated at 100°C for 5 min. The solution was centrifuged at 8,000 rpm for one min at room temperature and the supernatant was discarded. To the pellet, 250 ul of LB1 buffer was added and vortexed for 20 sec followed by 5 min incubation at room temperature. Buffer LB2 (250 ul) was added followed by 20ul of Proteinase K solution (20mg/ml). The solution was mixed and incubated at 65°C for 20 min with intermittent vortex. To this 10ul of LB3 buffer was added and mixed, followed by incubation at 65°C for 10 min. The solution was then centrifuged at 12,000 rpm for 1 min and the resultant supernatant transferred to a fresh tube. To this an equal volume of Phenol:Chloroform was added and mixed followed by centrifugation at 12,000 rpm for 5 min. Carefully the aqueous phase was transferred to a fresh tube and 800ul of chilled WB2 buffer was added followed by mixing. The solution was centrifuged at 12,000 rpm for 20 min and the supernatant was discarded. To this, 400ul of chilled WB3 buffer was added and centrifuged at 12,000 rpm for 1 min. The supernatant was discarded and the pellet was air-dried. Finally 100ul of RE buffer was added and the DNA solution was stored at 2–8°C.

### Genome Sequencing, Spoligotyping and Drug sensitivity profiling

Briefly, 101 base paired-end DNA libraries were generated using Illumina paired-end sample preparation kit following standard manufacturer protocol [54]. 5 μg of genomic DNA (gDNA) was nebulized to generate double stranded DNA fragments of size less than 800 bp. The sticky ends of the fragmented DNA were converted to blunt ends using T4 DNA polymerase and Klenow enzyme and a single “A” base was added to 3′ end using polymerase activity of Klenow fragments (3′ to 5′ exo minus). To this “A” overhang, manufacturer specified paired end adapter with a single “T” overhang was ligated. Of these ligated products, ∼350 bp fragment was selected from a 2% agarose gel and selectively enriched by PCR using adapter specific primers. A minimum base quality of 30 was used for filtering the data. The DNA libraries were indexed and sequenced using the Illumina Genome Analyzer IIx platform following standard protocols provided by the manufacturer. The reads of newly sequenced genomes have been deposited in NCBI Sequence Read Archive (SRA) (http://www.ncbi.nlm.nih.gov/sra) with accessions SRR784917, SRR786188, SRR786373, SRR786397, SRR786667, SRR786668, SRR786669 and SRR786670 [52]. Spoligotyping and drug sensitivity profiling were performed using standard protocol and spoligotyping kit from Ocimum Biosolutions Ltd. by Premas Biotech, India.

### *De-novo* Assembly

*De-novo* assemblies were performed for the eight Indian OSDD isolates sequenced in the study. Raw paired-end reads obtained after sequencing were *de novo* assembled using Velvet software [55] using different hash lengths (from 11 to 63). A 10 fold coverage cut-off was used for each hash length, and the best assembly for each of the strain was selected based on the highest N50 values (N50 denotes the weighted median statistic meaning that 50% of the entire assembly is contained in contigs or scaffolds equal to or larger than this value) and also the total assembly size. It was observed that assemblies with higher N50 values and assembly size tend to produce a decent and reliable assembled genome. Only contigs greater than 300bp were included in the final assembly output for further analysis. The contig assemblies are available at http://genome.igib.res.in/Mtb_Pangenome.

### Gene Prediction and Translation

Gene prediction and their corresponding protein translations were performed for all the 96 Mtb strains using Prodigal (version 2.5) which has improved translation initiation site recognition capability and produces less number of false positives [56]. Mtb gene prediction by Prodigal has been reported to be better than or equivalent to commonly used gene prediction algorithms [56]. WGS of the reference MTBC genomes and scaffolds/contigs of draft genomes were used to predict their CDS. We used Prodigal in normal mode with default parameters. Genome sequences of each strain was provided as input and gene predictions along with its CDS translations were obtained as output using standard command line options. We didn’t allow the predicted genes to run off the edges of the contigs. The raw gene predictions are available at http://genome.igib.res.in/Mtb_Pangenome/Pred_genes.

### Homologous gene clustering

We analysed the genomes using homologous clustering approach. Clustering tasks were performed with the tool CD-HIT [57], using an empirical threshold of 70% global sequence identity which is calculated as number of identical amino acids in alignment divided by full length of the shorter sequence. Orthologous clusters were determined by selecting HGCs which had only one sequence from each strain. If a HGC had more than one sequence from same strain it was considered a paralog cluster.

### Core-gene tree

A core-gene tree was calculated for all the 96 MTBC strains using the 100% core (i.e. hard-core) protein sequences using the approach followed by Kaas *et al* [47]. Briefly, in order to create the core-gene tree, we used only orthologous clusters. A multiple sequence alignment of the protein sequences for each cluster was done using MUSCLE version 3.8.31 [58]. The alignments were concatenated using FASconCAT [59]. Bootstrapping was done to generate 500 resamples using Seqboot version 3.695 [60]. Distance matrices were calculated for both original alignment as well as bootstrapped alignment using protdist version 3.695 [60]. Trees for both the original and bootstrapped matrices were determined by FastME available from NCBI [61]. FastME is based on the minimum evolution method and has been shown to have better accuracy than other commonly used methods such as the Neighbor joining (NJ) [61]. A comparative study of trees obtained from original alignment and resampled alignment was done by CompareToBootstrap [62]. The final un-rooted core-gene tree was visualized in FigTree (http://tree.bio.ed.ac.uk/software/figtree/).

### Evolution of Core and accessory genome size

We also estimated the change in the sizes of core and accessory gene content of MTBC each time a new strain is added to the analysis. For this we developed exponential decay (for core component) and exponential growth (for accessory component) models using MATLAB (version R2009b). We first generated random combinations (limiting it to 100, to avoid the combinatorial explosion) of genomes being sampled each time (i.e. 1, 2, 3, 4, …..96) using a bespoke Perl script followed by counting the presence or absence of genes in these combinations. Since the aim was to observe the change in size of the core and accessory genome with each new genome added to the analysis and to decide the saturation levels, we plotted the exponential models of the median values of the counts obtained as has been reported previously also [63].

### Pangenome estimation and annotation

Core and Pangenome estimation was performed by clustering all the translated CDS sequences of the 96 Mtb strains. The representative sequences of all clusters were then subjected to annotation using BLAST2GO [64]. The BLAST2GO basically performs annotation in three main steps: Firstly, it performs BLAST searches to find similar sequences for the input dataset using NCBI BLAST; Secondly, the program extracts the GO terms associated to each of the obtained BLAST hits using BLAST hit accessions using four different mappings and at last, returns an evaluated GO annotation for the query sequence(s) using an annotation rule [64]. Data mining on the results were carried out using custom Perl scripts. Data was parsed so as to reveal core and accessory genome clusters, clusters unique to each strain, clusters shared among a given strain pair, clusters of genes (pertaining to their absence in laboratory strains, hence called novel) present in greater than 1/3^rd^ of the clinical isolates and absent from the laboratory strains.

### Gene sequences specific to clinical strains

From the accessory gene clusters, we filtered clusters which were absent in the laboratory strains, Mtb H37Rv and Mtb H37Ra, but were present in more than 1/3^rd^ of the clinical strains being considered. The clusters were further matched against the laboratory whole genome sequences using BLAT [36], so as to reveal the level of similarity if any, in the laboratory strains.

### Validation of genes by PCR

Some of the genes identified from the newly sequenced strains were validated using PCR amplification followed by di-deoxy chain termination sequencing. List of validated genes, primer pairs and annealing temperature used for PCR has been summarized in S2 Table. PCR primers were designed using the NCBI primer BLAST [37]. Apart from 8 clinical isolates obtained from OSDD repository we have procured the *Mycobacterium tuberculosis* reference strains (H37RV—ATCC 25618D-5 and H37Ra—ATCC 25177D-5) from ATCC for the PCR validation. PCR mix constituted by 5 μl of 10X *Taq* buffer (Fermentas), 3 μl of 25mM MgCl2 (Fermentas), 2μl 10mM dNTP (Fermentas), 1 μl of respective forward and reverse primers (10mM), 36μl nuclease free water (Ambion) and 5 units *Taq* (Fermentas) along with 1 μl of respective sample DNA. Thermal cycling was performed by a PTC 200 thermal cycler (Biorad) with an initial denaturation by 94° for 5 minutes followed by 30 cycles of 94° for 45 seconds, respective annealing temperature as per S2 Table for 45 seconds, 72° for 45 seconds and a final extension of 72° for 10 minutes. The resultant PCR product was checked in 2% agarose 1X TAE gel and thereafter performed di-deoxy chain termination sequencing as originally described by Sanger *et al* [65].

## Supporting Information

S1 Fig Spoligotyping and Drug Susceptibility testing results. Drug sensitivity performed on a panel of 12 drugs and spoligotyping results for the eight OSDD strains.
(TIF)

S2 Fig Describing the soft-core. Essential gene based criteria for setting threshold for defining soft-core. We identified the number of overlapping essential genes by merging the three independent studies done on *Mycobacterium tuberculosis* H37Rv (Mtb). The study resulted in 348 genes shared amongst the three studies. After performing a BLAST search, 2,149 sequences out of the total 8,099 representative sequences matched with essential genes of Mtb. Out of the 2,149 BLAST hits 597 genes matched with the 348 overlapping genes. To set an optimal threshold for defining the soft-core we looked out for maximum number of essential genes matching with the core. The most optimal cut-off was found to be at 95% after which there was no significant rise in the number of essential genes falling in the core component and also assuming that all the 348 genes will be present at least once in the 95% core (i.e. 485 genes shared with BLAST hits).
(TIF)

S3 Fig Core gene tree with bootstrapped values obtained after comparing original tree with resampled trees. (TIF)

S4 Fig Distribution of essential genes of Mtb based on COG category. (TIF)

S5 Fig The number of sequences in each GO term for the GO category. biological process.
(TIF)

S6 Fig The number of sequences in each GO term for the GO category. cellular component.
(TIF)

S1 File Gene 4 alignment with reference genomes H37Rv and H37Ra. (DOCX)

S2 File Gene 8 alignment with reference genomes H37Rv and H37Ra. (DOCX)

S1 Table Strains description. Detailed description of strains used in current study.
(DOCX)

S2 Table Primers used. List of forward and reverse primers used for the validation and their expected product sizes.
(DOCX)
