# Supplementary figures and images for: Comparative Whole-Genome Analysis of Clinical Isolates Reveals Characteristic Architecture of Mycobacterium tuberculosis Pangenome
Source: PLoS One. 2015 Apr 8;10(4):e0122979. doi: 10.1371/journal.pone.0122979 (PMC4390332; doi:10.1371/journal.pone.0122979)

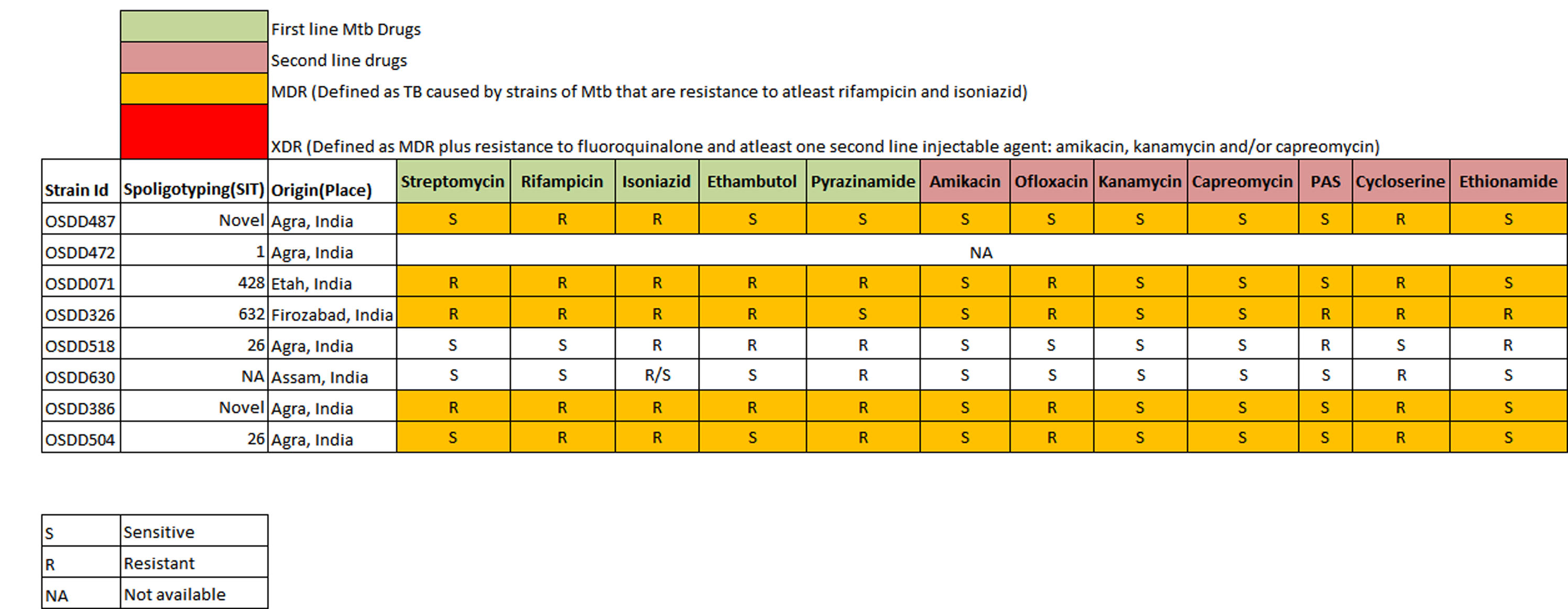

Supplement: S1 Fig — Drug sensitivity performed on a panel of 12 drugs and spoligotyping results for the eight OSDD strains. (TIF) [file pone.0122979.s001.tif]

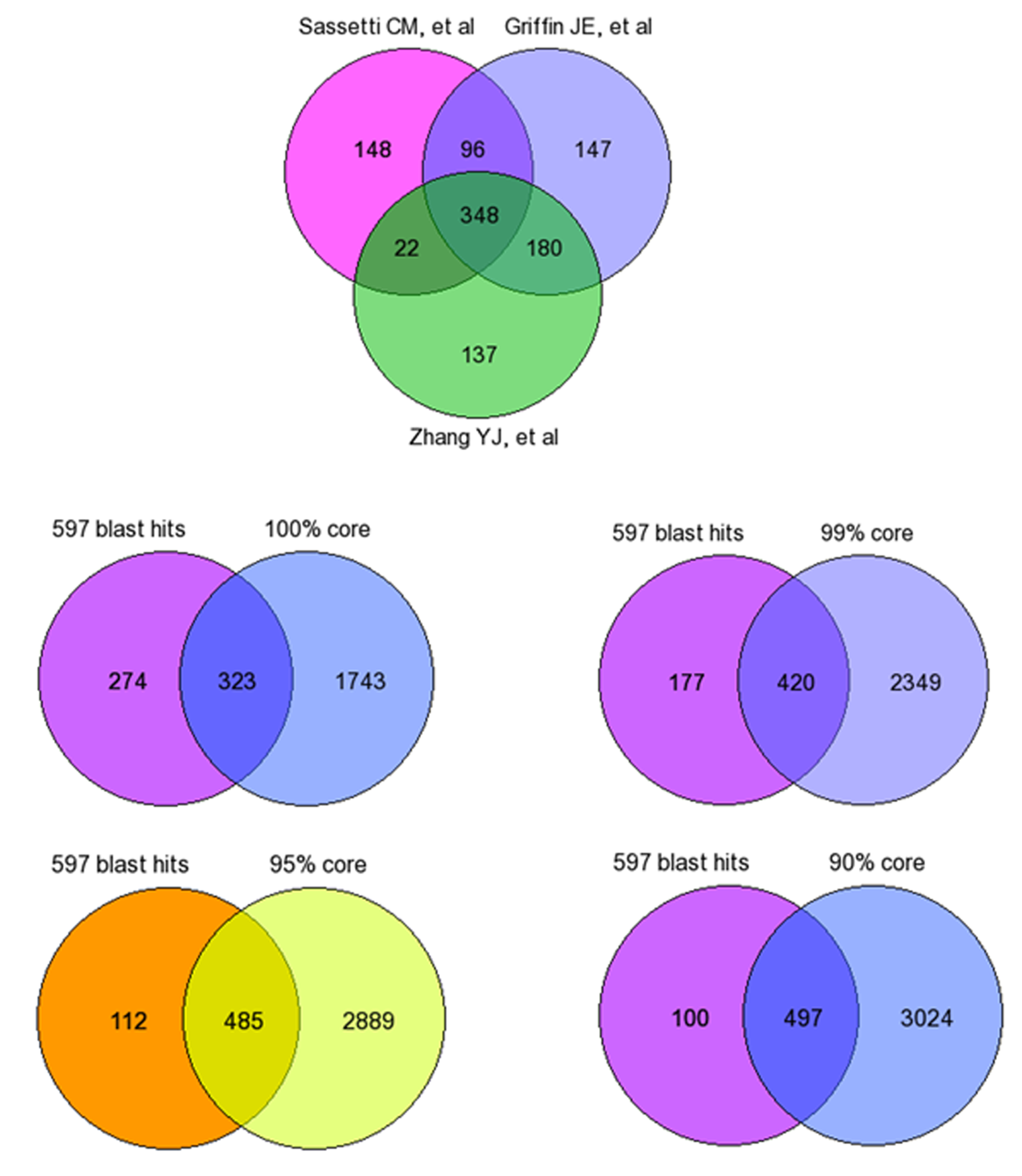

Supplement: S2 Fig — Essential gene based criteria for setting threshold for defining soft-core. We identified the number of overlapping essential genes by merging the three independent studies done on Mycobacterium tuberculosis H37Rv (Mtb). The study resulted in 348 genes shared amongst the three studies. After performing a BLAST search, 2,149 sequences out of the total 8,099 representative sequences matched with essential genes of Mtb. Out of the 2,149 BLAST hits 597 genes matched with the 348 overlapping genes. To set an optimal threshold for defining the soft-core we looked out for maximum number of essential genes matching with the core. The most optimal cut-off was found to be at 95% after which there was no significant rise in the number of essential genes falling in the core component and also assuming that all the 348 genes will be present at least once in the 95% core (i.e. 485 genes shared with BLAST hits). (TIF) [file pone.0122979.s002.tif]

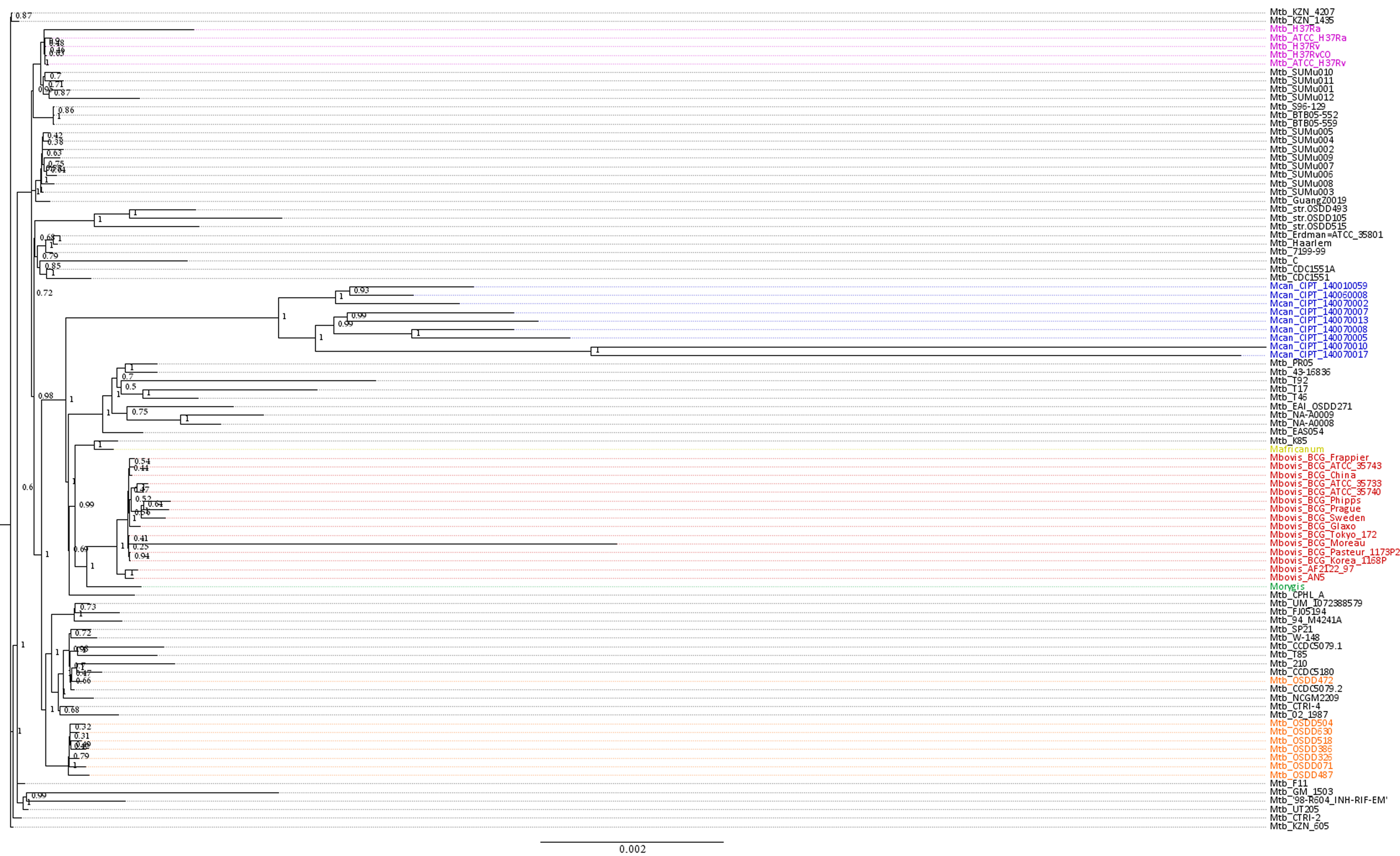

Supplement: S3 Fig — (TIF) [file pone.0122979.s003.tif]

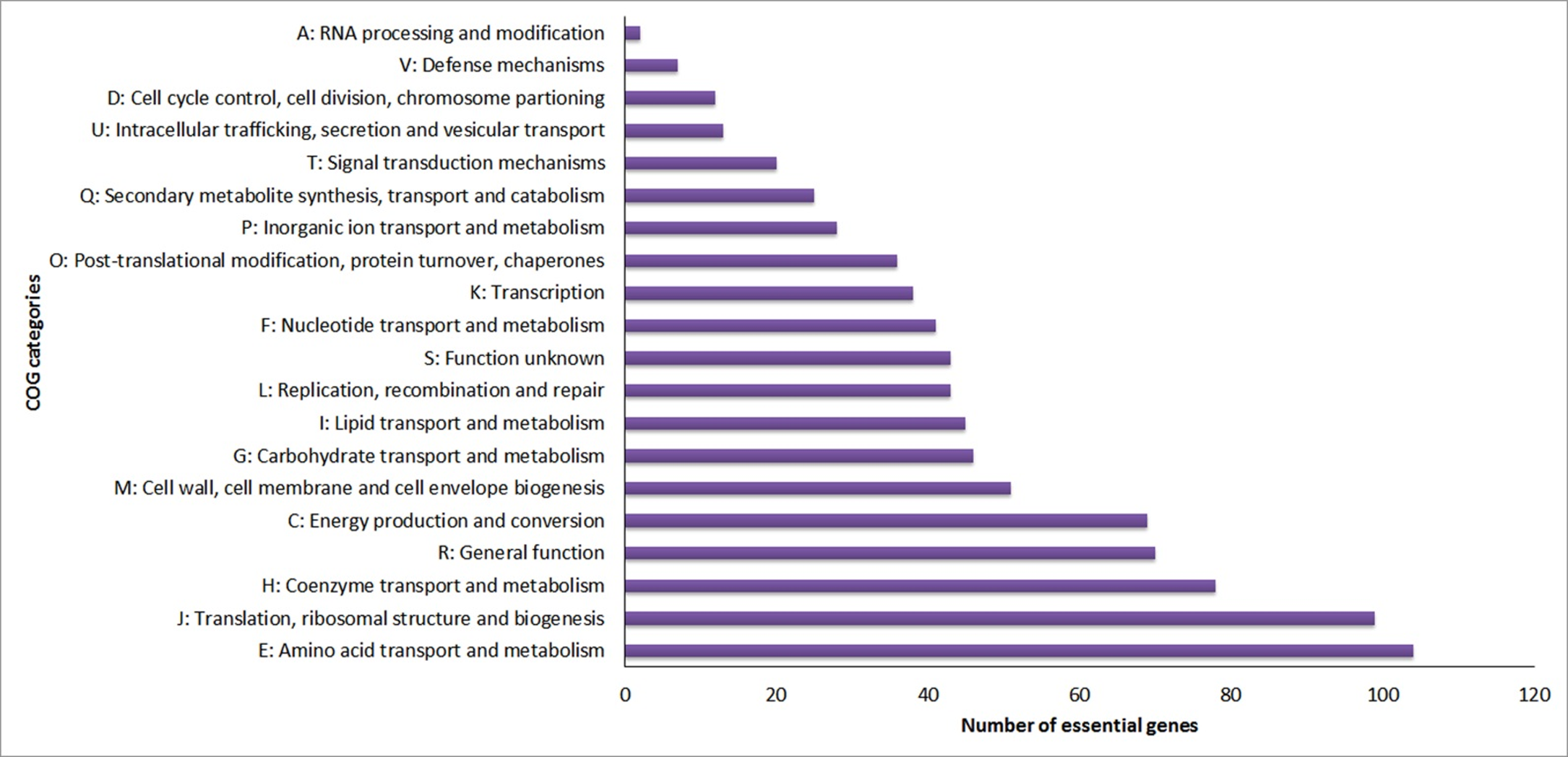

Supplement: S4 Fig — (TIF) [file pone.0122979.s004.tif]

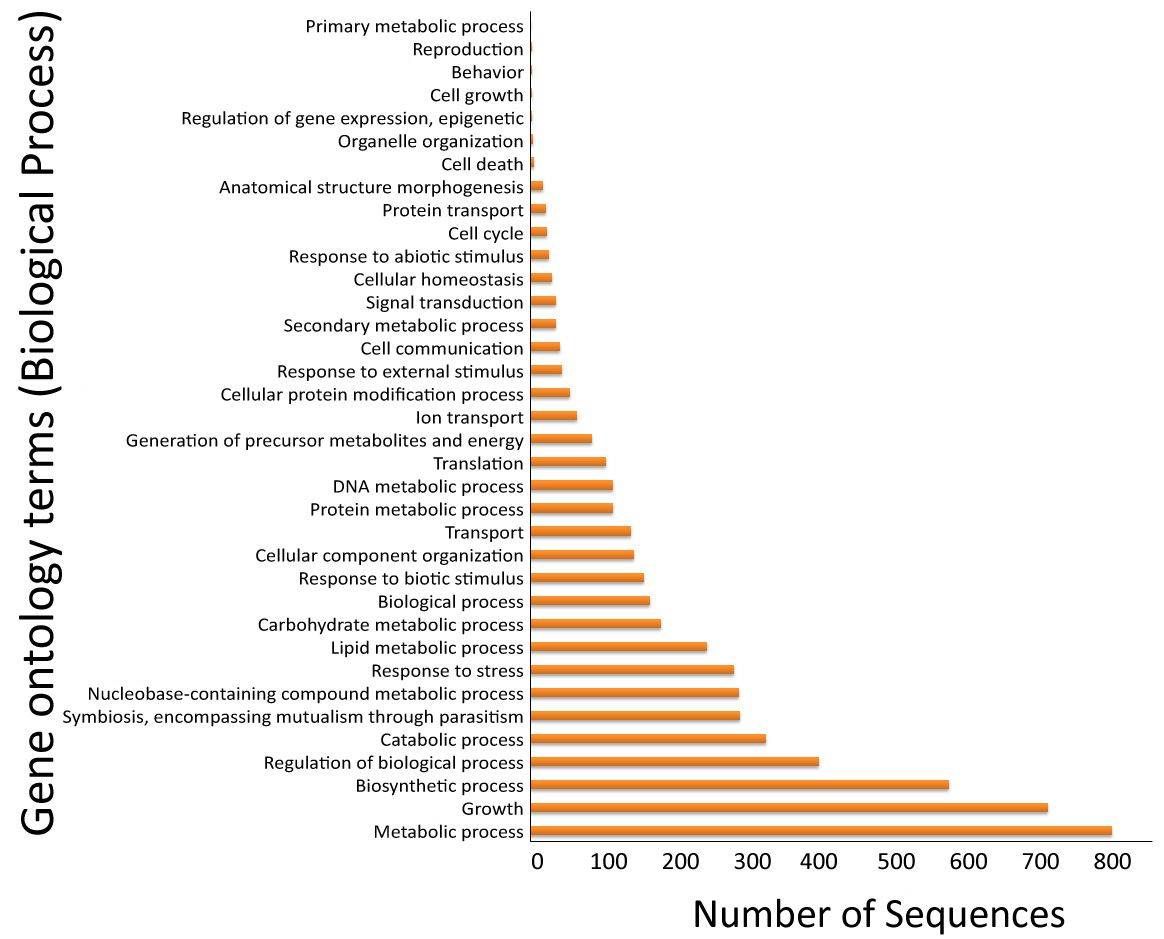

Supplement: S5 Fig — biological process. (TIF) [file pone.0122979.s005.tif]

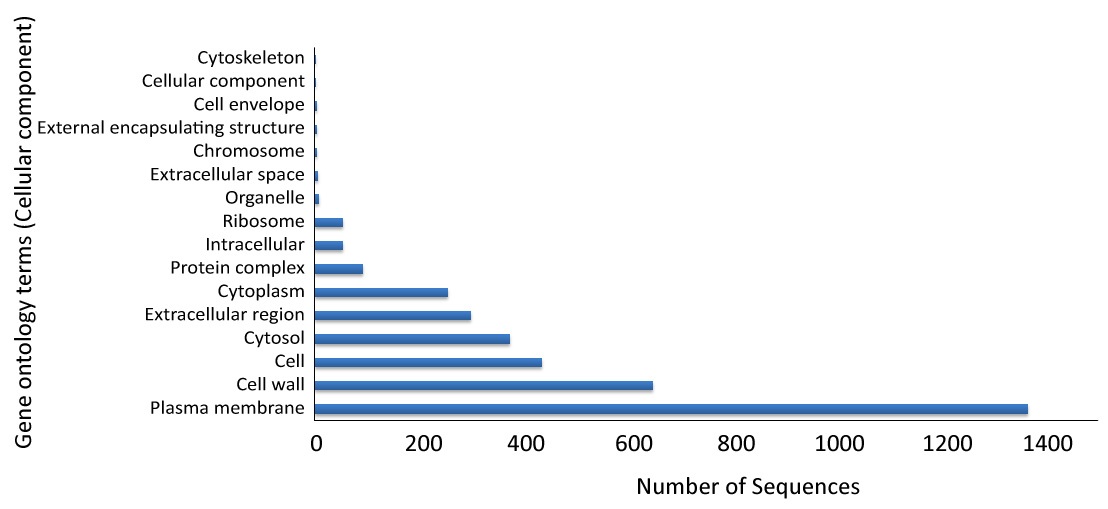

Supplement: S6 Fig — cellular component. (TIF) [file pone.0122979.s006.tif]
