# Supplementary material for: Comparative Whole-Genome Analysis of Clinical Isolates Reveals Characteristic Architecture of Mycobacterium tuberculosis Pangenome
Source: PLoS One. 2015 Apr 8;10(4):e0122979. doi: 10.1371/journal.pone.0122979 (PMC4390332; doi:10.1371/journal.pone.0122979)
Supplement: S1 File — (DOCX) [file pone.0122979.s007.docx]

**S1 file Gene 4 alignment with reference genomes H37Rv and H37Ra**


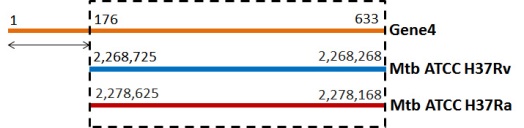


**Figure A** Schematic representation of alignment of Gene 4 with reference genomes H37Rv and H37Ra showing 175bp insertion in Gene 4. Below is base by base alignment of the gene product with H37Rv and H37Ra. Additionally, the PCR product of Gene 4 (with strain OSDD472) and H37Rv and H37Ra was sequenced using the classical dideoxy chain termination method.

**
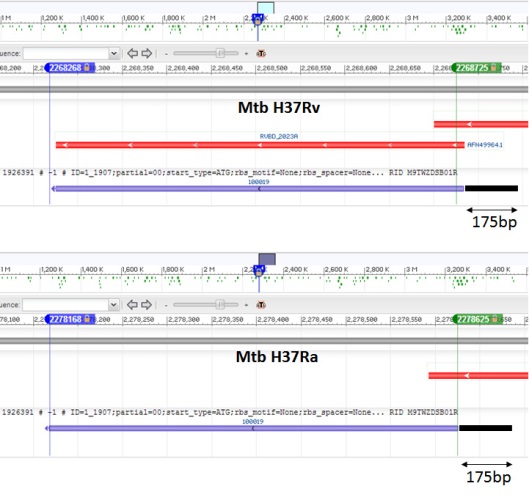
**

**Figure B** The figure shows a snapshot of the genomic region encompassing the loci where Gene 4 shares similarity with H37Rv and H37Ra. The region has been annotated as a hypothetical protein in H37Rv genome along the complementary strand (red color), which also explains the appearance of differently sized product in H37Rv. Alignment of the sequence of the PCR product of Gene 4 is shown in purple color. A small proportion of a hypothetical protein (red color) overlapping in H37Ra could possibly explain the appearance of differently sized product in H37Ra. The black line denotes additional 175bp found in Gene 4 and absent from H37Rv and H37Ra.

**Text A The predicted ORF Sequence of Gene 4 in OSDD strains (Length = 633bp, PCR product size = 496bp):**

ATGACAAGCGTCGCTCAGCTAGAGCACTATCTAGAGGAGCACTTGACCAAAGAGCTGGCATGGTTATTGCGTGCT**gcaacagaatggcacgcgca**ACATTGTATGAATCTTGGGATTGACGGCTATAGCATGCAGGTCTACGCACTCGATTCGACTGTGCTGCACGCCCGGACGCTGTTTGAATTCTTCACGCAGAATACAAGCGTAGGACAAAACGCGAATTACTACAATTGCACTGTGTACAAGGTACCGCTTATCGGATCGATCTTGTATGAATTTCACTGGAGAAGGCCGATTCATAGTCATATGATGCACGCGCAAGATAGGCGGCCGGTTACACAGTTACCAACATATGACGACCACGCGCAAACAAAACCGTTGAATGAGATGCCGGTGGACTTTGCCAAAGAGATAGTACGTTTGTGGCGCGTGTTCGTCAAAGATTTAAATAATCATACAAATTTACATTTTCGGCCAATCGGCGCTACAGCACAGACGGCGTTGGCATCTGAAATCAATGCCGCTAAAAGAGTCCGTACAAATGACGTT**acgcaacgtcagatcgctgtcg**GAAAGGAAACGAGCAGGCTGGAACCGAATTTTTCGATTCCGCAGATTGAATGGCCTGCCTAG

**Text B Sequences of the PCR product of Gene 4 (strain-OSDD472) and H37Rv and H37Ra as determined by Dideoxy chain termination method:**

**PCR Product Sequenced by Dideoxy chain termination sequencing: Mtb H37Rv by Gene-4 primer**

**AGATGGGCGGGTCCAGTTACCACATATGACGACCACGCGCAAACAAAACCGTTGAATGAGATGCCGGTGGACTTTGCCAAAGAGATAGTACGTTTGTGGCGCGTGTTCGTCAAAGATTTAAATAATCATACAAATTTACAGTTTCGGCCAATCGGCGCTACAGCACAGACGGCGTTGGCATCTGAAATCAATGCCGCTAAAAGAGTCCGTACAAATGACGTTACGCAACGTCAGATCGCTGTCGA**

**PCR Product Sequenced by Dideoxy chain termination sequencing: Mtb H37Ra by Gene-4 primer**

**AGAAGGCGGTAAAGTTACCACATATGACGACCACGCGCAAACAAAACCGTTGAATGAGATGCCGGTGGACTTTGCCAAAGAGATAGTACGTTTGTGGCGCGTGTTCGTCAAAGATTTAAATAATCATACAAATTTACAGTTTCGGCCAATCGGCGCTACAGCACAGACGGCGTTGGCATCTGAAATCAATGCCGCTAAAAGAGTCCGTACAAATGACGTTACGCAACGTCAGATCGCTGTCGA**

**PCR Product Sequenced by Dideoxy chain termination sequencing: OSDD 472 by Gene-4 primer**

**AAGTTAGGATCCTGGGATGACGGCTATAGCATGCAGGTCTACGCACTCGATTCGACTGTGCTGCACGCCCGGACGCTGTTTGAATTCTTCACGCAGAATACAAGCGTAGGACAAAACGCGAATTACTACAATTGCACTGTGTACAAGGTACCGCTTATCGGATCGATCTTGGTATCAATTTCACTGGAGAAGGCCGATTCATAGTCATATGATGCACGCGCAAGATAGGCGGCCGGTTACACAGTTACCAACATATGACGACCACGCGCAAACAAAACCGTTGAATGAGATGCCGGTGGACTTTGCCAAAGAGATAGTACGTTTGTGGCGCGTGTTCGTCAAAGATTTAAATAATCATACAAATTTACAGTTTCGGCCAATCGGCGCTACAGCACAGACGGCGTTGGCATCTGAAATCAATGCCGCTAAAAGAGTCCGTACAAATGACGTTACGCAACGCG**

**Text C Alignment of predicted ORF of Gene 4 sequence with H37RV**

Query: Gene 4, Sbjct: H37Rv, Forward and Reverse Primers: Lower case (red colored),

Insertion Sequence: Red colored

Query 1 ATGACAAGCGTCGCTCAGCTAGAGCACTATCTAGAGGAGCACTTGACCAAAGAGCTGGCA 60

||||||||||||||||||||||||||||||||||||||||||||||||||||||||||||

Sbjct ------------------------------------------------------------

Query 61 TGGTTATTGCGTGCT**gcaacagaatggcacgcgca**ACATTGTATGAATCTTGGGATTGAC 120

||||||||||||||||||||||||||||||||||||||||||||||||||||||||||||

Sbjct ------------------------------------------------------------

Query 121 GGCTATAGCATGCAGGTCTACGCACTCGATTCGACTGTGCTGCACGCCCGGACGC 175

|||||||||||||||||||||||||||||||||||||||||||||||||||||||

Sbjct -------------------------------------------------------

Query 176 TGTTTGAATTCTTCACGCAGAATACAAGCGTAGGACAAAACGCGAATTACTACAATTGCA 235

||||||||||||||||||||||||||||||||||||||||||||||||||||||||||||

Sbjct 2268725 TGTTTGAATTCTTCACGCAGAATACAAGCGTAGGACAAAACGCGAATTACTACAATTGCA 2268666

Query 236 CTGTGTACAAGGTACCGCTTATCGGATCGATCTTGTATCAATTTCACTGGAGAAGGCCGA 295

||||||||||||||||||||||||||||||||||||||||||||||||||||||||||||

Sbjct 2268665 CTGTGTACAAGGTACCGCTTATCGGATCGATCTTGTATCAATTTCACTGGAGAAGGCCGA 2268606

Query 296 TTCATAGTCATATGATGCACGCGCAAGATAGGCGGCCGGTTACACAGTTACCAACATATG 355

||||||||||||||||||||||||||||||||||||||||||||||||||||||||||||

Sbjct 2268605 TTCATAGTCATATGATGCACGCGCAAGATAGGCGGCCGGTTACACAGTTACCAACATATG 2268546

Query 356 ACGACCACGCGCAAACAAAACCGTTGAATGAGATGCCGGTGGACTTTGCCAAAGAGATAG 415

||||||||||||||||||||||||||||||||||||||||||||||||||||||||||||

Sbjct 2268545 ACGACCACGCGCAAACAAAACCGTTGAATGAGATGCCGGTGGACTTTGCCAAAGAGATAG 2268486

Query 416 TACGTTTGTGGCGCGTGTTCGTCAAAGATTTAAATAATCATACAAATTTACAGTTTCGGC 475

||||||||||||||||||||||||||||||||||||||||||||||||||||||||||||

Sbjct 2268485 TACGTTTGTGGCGCGTGTTCGTCAAAGATTTAAATAATCATACAAATTTACAGTTTCGGC 2268426

Query 476 CAATCGGCGCTACAGCACAGACGGCGTTGGCATCTGAAATCAATGCCGCTAAAAGAGTCC 535

||||||||||||||||||||||||||||||||||||||||||||||||||||||||||||

Sbjct 2268425 CAATCGGCGCTACAGCACAGACGGCGTTGGCATCTGAAATCAATGCCGCTAAAAGAGTCC 2268366

Query 536 GTACAAATGACGTT**acgcaacgtcagatcgctgtcg**GAAAGGAAACGAGCAGGCTGGAAC 595

||||||||||||||||||||||||||||||||||||||||||||||||||||||||||||

Sbjct 2268365 GTACAAATGACGTT**acgcaacgtcagatcgctgtcg**GAAAGGAAACGAGCAGGCTGGAAC 2268306

Query 596 CGAATTTTTCGATTCCGCAGATTGAATGGCCTGCCTAG 633

||||||||||||||||||||||||||||||||||||||

Sbjct 2268305 CGAATTTTTCGATTCCGCAGATTGAATGGCCTGCCTAG 2268268

**Text D Alignment of predicted ORF of Gene 4 sequence with H37Ra**

Query: Gene 4, Sbjct: H37Ra, Forward and Reverse Primers: Lower case (red colored),

Insertion Sequence: Red colored

Query 1 ATGACAAGCGTCGCTCAGCTAGAGCACTATCTAGAGGAGCACTTGACCAAAGAGCTGGCA 60

||||||||||||||||||||||||||||||||||||||||||||||||||||||||||||

Sbjct ------------------------------------------------------------

Query 61 TGGTTATTGCGTGCT**gcaacagaatggcacgcgca**ACATTGTATGAATCTTGGGATTGAC 120

||||||||||||||||||||||||||||||||||||||||||||||||||||||||||||

Sbjct ------------------------------------------------------------

Query 121 GGCTATAGCATGCAGGTCTACGCACTCGATTCGACTGTGCTGCACGCCCGGACGC 175

|||||||||||||||||||||||||||||||||||||||||||||||||||||||

Sbjct -------------------------------------------------------

|  |  |  |  |  |
| --- | --- | --- | --- | --- |
|  |  |  |  |  |

Query 176 TGTTTGAATTCTTCACGCAGAATACAAGCGTAGGACAAAACGCGAATTACTACAATTGCA 235

||||||||||||||||||||||||||||||||||||||||||||||||||||||||||||

Sbjct 2278625 TGTTTGAATTCTTCACGCAGAATACAAGCGTAGGACAAAACGCGAATTACTACAATTGCA 2278566

Query 236 CTGTGTACAAGGTACCGCTTATCGGATCGATCTTGTATCAATTTCACTGGAGAAGGCCGA 295

||||||||||||||||||||||||||||||||||||||||||||||||||||||||||||

Sbjct 2278565 CTGTGTACAAGGTACCGCTTATCGGATCGATCTTGTATCAATTTCACTGGAGAAGGCCGA 2278506

Query 296 TTCATAGTCATATGATGCACGCGCAAGATAGGCGGCCGGTTACACAGTTACCAACATATG 355

||||||||||||||||||||||||||||||||||||||||||||||||||||||||||||

Sbjct 2278505 TTCATAGTCATATGATGCACGCGCAAGATAGGCGGCCGGTTACACAGTTACCAACATATG 2278446

Query 356 ACGACCACGCGCAAACAAAACCGTTGAATGAGATGCCGGTGGACTTTGCCAAAGAGATAG 415

||||||||||||||||||||||||||||||||||||||||||||||||||||||||||||

Sbjct 2278445 ACGACCACGCGCAAACAAAACCGTTGAATGAGATGCCGGTGGACTTTGCCAAAGAGATAG 2278386

Query 416 TACGTTTGTGGCGCGTGTTCGTCAAAGATTTAAATAATCATACAAATTTACAGTTTCGGC 475

||||||||||||||||||||||||||||||||||||||||||||||||||||||||||||

Sbjct 2278385 TACGTTTGTGGCGCGTGTTCGTCAAAGATTTAAATAATCATACAAATTTACAGTTTCGGC 2278326

Query 476 CAATCGGCGCTACAGCACAGACGGCGTTGGCATCTGAAATCAATGCCGCTAAAAGAGTCC 535

||||||||||||||||||||||||||||||||||||||||||||||||||||||||||||

Sbjct 2278325 CAATCGGCGCTACAGCACAGACGGCGTTGGCATCTGAAATCAATGCCGCTAAAAGAGTCC 2278266

Query 536 GTACAAATGACGTT**acgcaacgtcagatcgctgtcg**GAAAGGAAACGAGCAGGCTGGAAC 595

||||||||||||||||||||||||||||||||||||||||||||||||||||||||||||

Sbjct 2278265 GTACAAATGACGTT**acgcaacgtcagatcgctgtcg**GAAAGGAAACGAGCAGGCTGGAAC 2278206

Query 596 CGAATTTTTCGATTCCGCAGATTGAATGGCCTGCCTAG 633

||||||||||||||||||||||||||||||||||||||

Sbjct 2278205 CGAATTTTTCGATTCCGCAGATTGAATGGCCTGCCTAG 2278168
