# Supplementary material for: Comparative Whole-Genome Analysis of Clinical Isolates Reveals Characteristic Architecture of Mycobacterium tuberculosis Pangenome
Source: PLoS One. 2015 Apr 8;10(4):e0122979. doi: 10.1371/journal.pone.0122979 (PMC4390332; doi:10.1371/journal.pone.0122979)
Supplement: S2 File — (DOCX) [file pone.0122979.s008.docx]

**S2 File Gene 8 alignment with reference genomes H37Rv and H37Ra**

**
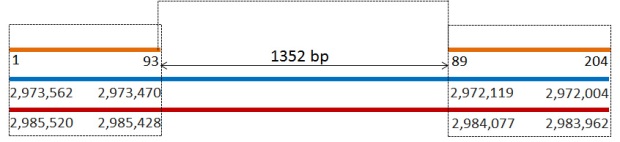
**

**Figure A** Schematic representation of alignment of Gene 8 against reference genomes H37Rv and H37Ra showing 1,356bp deletion in Gene 8. Below is base by base alignment of the gene product with H37Rv and H37Ra. The deleted region of 1352bp in Gene8 is annotated as repeat region in H37Rv and H37Ra genomes, showing the presence of IS6110 repeat element. Additionally, the PCR product of Gene 8 was sequenced using the classical dideoxy chain termination method for OSDD472 strain.


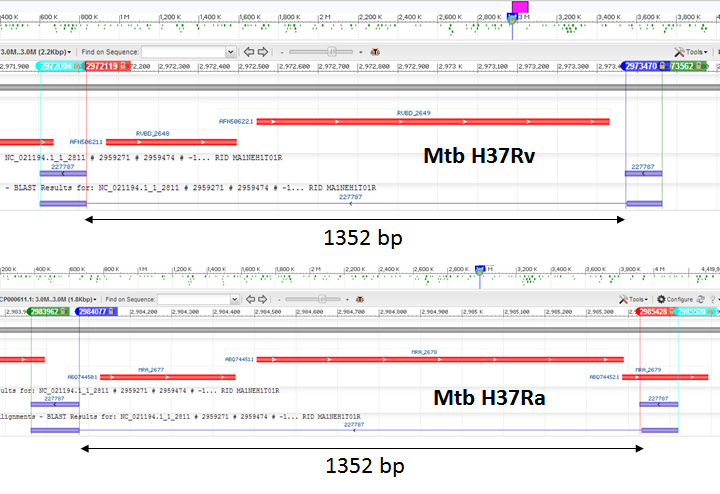


**Figure B** Snapshot of genomic loci in H37Rv and H37Ra genomes where Gene 8 aligns majorly consists of a repeat region. The 1,352bp sequence region absent from Gene 8 corresponds to an IS element which is annotated as a probable transposase. The purple colored regions indicate the Gene 8 sequence aligned with the reference genomes and a 1,356bp sequence deletion in OSDD strains.

**Text A The predicted ORF Sequence of Gene 8 in OSDD strains (Length = 201bp, PCR product size = 195bp):**

ATGGgtgtgcagcgcgcgattgagAGGGAGATTCGGCGTCGCTATGAGGCTTGGCGCCGCCAGGCTGAGCAGGCACGCAAGGCCGCCGAAGATGAGCTGCGCAAGCGTGGACGTGAGCGTCGCCCGTTGGACAAGTTGCCGCCGGGGCCCATCCCGGGCACCGGCGGTCAGCCGCTccaaccgtttaaaccatcccggtAG

**Text B Sequences of the PCR product of Gene 8 (strain-OSDD472) as determined by Dideoxy chain termination method:**

**PCR Product Sequenced by Dideoxy chain termination sequencing: OSDD 472 by Gene-8 primer**

AGGGAGATTCGGCGTCGCTATGAGGCTTGGCGCCGCCAGGCTGAGCAGGCACGCAAGGCCGCCGAAGATGAGCTGCGCAAGCGTGGACGTGAGCGTCGCCCGTTGGACAAGTTGCCGCCGGGGCCCATCCCGGGCACCGGCGGTCAGCCGCTCCAACCGTTTAAACCATCCCGGT

**Text C Alignment of predicted ORF of Gene 8 sequence with H37RV**

Query: Gene 8, Sbjct: H37Rv, Forward and Reverse Primers: Lower case (red colored),

Deletion Sequence: Blue colored, Overlapping loci: Green colored

Query 1 ATGGgtgtgcagcgcgcgattgagAGGGAGATTCGGCGTCGCTATGAGGCTTGGCGCCGC 60

||||||||||||||||||||||||||||||||||||||||||||||||||||||||||||

Sbjct 2973562 ATGGgtgtgcagcgcgcgattgagAGGGAGATTCGGCGTCGCTATGAGGCTTGGCGCCGC 2973503

Query 61 CAGGCTGAGCAGGCACGCAAGGCCGCCGACGAA 93

|||||||||||||||||||||||||||||||||

Sbjct 2973502 CAGGCTGAGCAGGCACGCAAGGCCGCCGATGAA 2973470

Query ------------------------------------------------------------

||||||||||||||||||||||||||||||||||||||||||||||||||||||||||||

Sbjct 2973469 ATGAACCGCCCCGGTGAGTCCGGAGACTCTCTGATCTGAGACCTCAGCCGGCGGCTGGTC 2973410

Query ------------------------------------------------------------

||||||||||||||||||||||||||||||||||||||||||||||||||||||||||||

Sbjct 2973409 TCTGGCGTTGAGCGTAGTAGGCAGCCTCGAGTTCGACCGGCGGGACGTCGCCGCAGTACT 2973350

Query ------------------------------------------------------------

||||||||||||||||||||||||||||||||||||||||||||||||||||||||||||

Sbjct 2973349 GGTAGAGGCGGCGATGGTTGAACCAGTCGACCCAGCGCGCGGTGGCCAACTCGACATCCT 2973290

Query ------------------------------------------------------------

||||||||||||||||||||||||||||||||||||||||||||||||||||||||||||

Sbjct 2973289 CGATGGACCGCCAGGGCTTGCCGGGTTTGATCAGCTCGGTCTTGTATAGGCCGTTGATCG 2973230

Query ------------------------------------------------------------

||||||||||||||||||||||||||||||||||||||||||||||||||||||||||||

Sbjct 2973229 TCTCGGCTAGTGCATTGTCATAGGAGCTTCCGACCGCTCCGACCGACGGTTGGATGCCTG 2973170

Query ------------------------------------------------------------

||||||||||||||||||||||||||||||||||||||||||||||||||||||||||||

Sbjct 2973169 CCTCGGCGAGCCGCTCGCTGAACCGGATCGATGTGTACTGAGATCCCCTATCCGTATGGT 2973110

Query ------------------------------------------------------------

||||||||||||||||||||||||||||||||||||||||||||||||||||||||||||

Sbjct 2973109 GGATAACGTCTTTCAGGTCGAGTACGCCTTCTTGTTGGCGGGTCCAGATGGCTTGCTCGA 2973050

Query ------------------------------------------------------------

||||||||||||||||||||||||||||||||||||||||||||||||||||||||||||

Sbjct 2973049 TCGCGTCGAGGACCATGGAGGTGGCCATCGTGGAAGCGACCCGCCAGCCCAGGATCCTGC 2972990

Query ------------------------------------------------------------

||||||||||||||||||||||||||||||||||||||||||||||||||||||||||||

Sbjct 2972989 GAGCGTAGGCGTCGGTGACAAAGGCCACGTAGGCGAACCCTGCCCAGGTCGACACATAGG 2972930

Query ------------------------------------------------------------

||||||||||||||||||||||||||||||||||||||||||||||||||||||||||||

Sbjct 2972929 TGAGGTCTGCTACCCACAGCCGGTTAGGTGCTGGTGGTCCGAAGCGGCGCTGGACGAGAT 2972870

Query ------------------------------------------------------------

||||||||||||||||||||||||||||||||||||||||||||||||||||||||||||

Sbjct 2972869 CGGCGGGACGGGCTGTGGCCGGATCAGCGATCGTGGTCCTGCGGGCTTTGCCGCGGGTGG 2972810

Query ------------------------------------------------------------

||||||||||||||||||||||||||||||||||||||||||||||||||||||||||||

Sbjct 2972809 TCCCGGACAGGCCGAGTTTGGTCATCAGCCGTTCGACGGTGCATCTGGCCACCTCGATGC 2972750

Query ------------------------------------------------------------

||||||||||||||||||||||||||||||||||||||||||||||||||||||||||||

Sbjct 2972749 CCTCACGGTTCAGGGTTAGCCACACTTTGCGGGCACCGTAAACACCGTAGTTGGCGGCGT 2972690

Query ------------------------------------------------------------

||||||||||||||||||||||||||||||||||||||||||||||||||||||||||||

Sbjct 2972689 GGACGCGGCTGATGTGCTCCTTGAGTTCGCCATCGCGCAGCTCGCGGCGGCTGGGCTCCC 2972630

Query ------------------------------------------------------------

||||||||||||||||||||||||||||||||||||||||||||||||||||||||||||

Sbjct 2972629 GGTTGATGTGGTCGTAGTAGGTCGATGGGGCGATCGGCACACCCAGCTCGGTCAGCTGTG 2972570

Query ------------------------------------------------------------

||||||||||||||||||||||||||||||||||||||||||||||||||||||||||||

Sbjct 2972569 TGCAGATCGACTCGACACCCCACCGCAAACCATCGGGGCCCTCGCGGTGGCCCTGATGAT 2972510

Query ------------------------------------------------------------

||||||||||||||||||||||||||||||||||||||||||||||||||||||||||||

Sbjct 2972509 CGGCGATGAACCGGGTAATTAGCGTGCTGGCCGGTCGAGCTCGGCCGCGAAGAAAGCCGA 2972450

Query ------------------------------------------------------------

||||||||||||||||||||||||||||||||||||||||||||||||||||||||||||

Sbjct 2972449 CGCGGTCTTTAAAATCGCGTTCGCCCTTCGCAATTCGGCGTTGTCCCGCCGCAAGCGCTT 2972390

Query ------------------------------------------------------------

||||||||||||||||||||||||||||||||||||||||||||||||||||||||||||

Sbjct 2972389 CAGCTCAGCGGATTCTTCGGTCGTGGTCCCGGGCCGTGCGCCGGCATCGACCTGCGCCTG 2972330

Query ------------------------------------------------------------

||||||||||||||||||||||||||||||||||||||||||||||||||||||||||||

Sbjct 2972329 GCGCACCCACTTACGCACCGTCTCCGCGCAGCCAACACCAAGTAGACGGGCGACCTCACT 2972270

Query ------------------------------------------------------------

||||||||||||||||||||||||||||||||||||||||||||||||||||||||||||

Sbjct 2972269 GATCGCTGCCCACTCCGAATCGTGCTGACCGCGGATCTCTGCGACCATCCGCACCGCCCG 2972210

Query ------------------------------------------------------------

||||||||||||||||||||||||||||||||||||||||||||||||||||||||||||

Sbjct 2972209 CTCACGCAGCTCCGGCGGGTACCTCCTCGATGAACCACCTGACATGACCCCATCCTTTCC 2972150

Query ------------------------------------

||||||||||||||||||||||||||||||||||||

Sbjct 2972149 AAGAACTGGAGTCTCCGGACATGCCGGGGCGGTTCA 2972118

Query 89 ACGAAGATGAGCTGCGCAAGCGTGGACGTGAGCGTCGCCCGTTGGACAAGTTGCCGCCGG 148

||||||||||||||||||||||||||||||||||||||||||||||||||||||||||||

Sbjct 2972119 ACGAAGATGAGCTGCGCAAGCGTGGACGTGAGCGTCGCCCGTTGGACAAGTTGCCGCCGG 2972060

Query 149 GGCCCATCCCGGGCACCGGCGGTCAGCCGCTccaaccgtttaaaccatcccggtAG 204

||||||||||||||||||||||||||||||||||||||||||||||||||||||||

Sbjct 2972059 GGCCCATCCCGGGCACCGGCGGTCAGCCGCTccaaccgtttaaaccatcccggtAG 2972004

**Text D Alignment of predicted ORF of Gene 8 sequence with H37Ra**

Query: Gene 8, Sbjct: H37Ra, Forward and Reverse Primers: Lower case (red colored),

Deletion Sequence: Blue colored, Overlapping loci: Green colored

Query 1 ATGGgtgtgcagcgcgcgattgagAGGGAGATTCGGCGTCGCTATGAGGCTTGGCGCCGC 60

||||||||||||||||||||||||||||||||||||||||||||||||||||||||||||

Sbjct 2985520 ATGGgtgtgcagcgcgcgattgagAGGGAGATTCGGCGTCGCTATGAGGCTTGGCGCCGC 2985461

Query 61 CAGGCTGAGCAGGCACGCAAGGCCGCCGACGAA 93

||||||||||||||||||||||||||||| |||

Sbjct 2985460 CAGGCTGAGCAGGCACGCAAGGCCGCCGATGAA 2985428

Query ------------------------------------------------------------

||||||||||||||||||||||||||||||||||||||||||||||||||||||||||||

Sbjct 2985427 CCGCCCCGGTGAGTCCGGAGACTCTCTGATCTGAGACCTCAGCCGGCGGCTGGTCTCTGG 2985367

Query ------------------------------------------------------------

||||||||||||||||||||||||||||||||||||||||||||||||||||||||||||

Sbjct 2985366 CGTTGAGCGTAGTAGGCAGCCTCGAGTTCGACCGGCGGGACGTCGCCGCAGTACTGGTAG 2985307

Query ------------------------------------------------------------

||||||||||||||||||||||||||||||||||||||||||||||||||||||||||||

Sbjct 2985306 AGGCGGCGATGGTTGAACCAGTCGACCCAGCGCGCGGTGGCCAACTCGACATCCTCGATG 2985247

Query ------------------------------------------------------------

||||||||||||||||||||||||||||||||||||||||||||||||||||||||||||

Sbjct 2985246 GACCGCCAGGGCTTGCCGGGTTTGATCAGCTCGGTCTTGTATAGGCCGTTGATCGTCTCG 2985187

Query ------------------------------------------------------------

||||||||||||||||||||||||||||||||||||||||||||||||||||||||||||

Sbjct 2985186 GCTAGTGCATTGTCATAGGAGCTTCCGACCGCTCCGACCGACGGTTGGATGCCTGCCTCG 2985127

Query ------------------------------------------------------------

||||||||||||||||||||||||||||||||||||||||||||||||||||||||||||

Sbjct 2985126 GCGAGCCGCTCGCTGAACCGGATCGATGTGTACTGAGATCCCCTATCCGTATGGTGGATA 2985067

Query ------------------------------------------------------------

||||||||||||||||||||||||||||||||||||||||||||||||||||||||||||

Sbjct 2985066 ACGTCTTTCAGGTCGAGTACGCCTTCTTGTTGGCGGGTCCAGATGGCTTGCTCGATCGCG 2985007

Query ------------------------------------------------------------

||||||||||||||||||||||||||||||||||||||||||||||||||||||||||||

Sbjct 2985006 TCGAGGACCATGGAGGTGGCCATCGTGGAAGCGACCCGCCAGCCCAGGATCCTGCGAGCG 2984947

Query ------------------------------------------------------------

||||||||||||||||||||||||||||||||||||||||||||||||||||||||||||

Sbjct 2984946 TAGGCGTCGGTGACAAAGCCCACGTAGGCGAACCCTGCCCAGGTCGACACATAGGTGAGG 2984887

Query ------------------------------------------------------------

||||||||||||||||||||||||||||||||||||||||||||||||||||||||||||

Sbjct 2984886 TCTGCTACCCACAGCCGGTTAGGTGCTGGTGGTCCGAAGCGGCGCTGGACGAGATCGGCG 2984827

Query ------------------------------------------------------------

||||||||||||||||||||||||||||||||||||||||||||||||||||||||||||

Sbjct 2984826 GGACGGGCTGTGGCCGGATCAGCGATCGTGGTCCTGCGGGCTTTGCCGCGGGTGGTCCCG 2984767

Query ------------------------------------------------------------

||||||||||||||||||||||||||||||||||||||||||||||||||||||||||||

Sbjct 2984766 GACAGGCCGAGTTTGGTCATCAGCCGTTCGACGGTGCATCTGGCCACCTCGATGCCCTCA 2984707

Query ------------------------------------------------------------

||||||||||||||||||||||||||||||||||||||||||||||||||||||||||||

Sbjct 2984706 CGGTTCAGGGTTAGCCACACTTTGCGGGCACCGTAAACACCGTAGTTGGCGGCGTGGACG 2984647

Query ------------------------------------------------------------

||||||||||||||||||||||||||||||||||||||||||||||||||||||||||||

Sbjct 2984646 CGGCTGATGTGCTCCTTGAGTTCGCCATCGCGCAGCTCGCGGCGGCTGGGCTCCCGGTTG 2984587

Query ------------------------------------------------------------

||||||||||||||||||||||||||||||||||||||||||||||||||||||||||||

Sbjct 2984586 ATGTGGTCGTAGTAGGTCGATGGGGCGATCGGCACACCCAGCTCGGTCAGCTGTGTGCAG 2984527

Query ------------------------------------------------------------

||||||||||||||||||||||||||||||||||||||||||||||||||||||||||||

Sbjct 2984526 ATCGACTCGACACCCCACCGCAAACCATCGGGGCCCTCGCGGTGGCCCTGATGATCGGCG 2984467

Query ------------------------------------------------------------

||||||||||||||||||||||||||||||||||||||||||||||||||||||||||||

Sbjct 2984466 ATGAACCGGGTAATTAGCGTGCTGGCCGGTCGAGCTCGGCCGCGAAGAAAGCCGACGCGG 2984407

Query ------------------------------------------------------------

||||||||||||||||||||||||||||||||||||||||||||||||||||||||||||

Sbjct 2984406 TCTTTAAAATCGCGTTCGCCCTTCGCAATTCGGCGTTGTCCCGCCGCAAGCGCTTCAGCT 2984347

Query ------------------------------------------------------------

||||||||||||||||||||||||||||||||||||||||||||||||||||||||||||

Sbjct 2984346 CAGCGGATTCTTCGGTCGTGGTCCCGGGCCGTGCGCCGGCATCGACCTGCGCCTGGCGCA 2984287

Query ------------------------------------------------------------

||||||||||||||||||||||||||||||||||||||||||||||||||||||||||||

Sbjct 2984286 CCCACTTACGCACCGTCTCCGCGCAGCCAACACCAAGTAGACGGGCGACCTCACTGATCG 2984227

Query ------------------------------------------------------------

||||||||||||||||||||||||||||||||||||||||||||||||||||||||||||

Sbjct 2984226 CTGCCCACTCCGAATCGTGCTGACCGCGGATCTCTGCGACCATCCGCACCGCCCGCTCAC 2984167

Query ------------------------------------------------------------

||||||||||||||||||||||||||||||||||||||||||||||||||||||||||||

Sbjct 2984166 GCAGCTCCGGCGGGTACCTCCTCGATGAACCACCTGACATGACCCCATCCTTTCCAAGAA 2984107

Query ------------------------------

||||||||||||||||||||||||||||||

Sbjct 2984106 CTGGAGTCTCCGGACATGCCGGGGCGGTTC 2984076

Query 89 ACGAAGATGAGCTGCGCAAGCGTGGACGTGAGCGTCGCCCGTTGGACAAGTTGCCGCCGG 148

||||||||||||||||||||||||||||||||||||||||||||||||||||||||||||

Sbjct 2984077 ACGAAGATGAGCTGCGCAAGCGTGGACGTGAGCGTCGCCCGTTGGACAAGTTGCCGCCGG 2984018

Query 149 GGCCCATCCCGGGCACCGGCGGTCAGCCGCTccaaccgtttaaaccatcccggtAG 204

||||||||||||||||||||||||||||||||||||||||||||||||||||||||

Sbjct 2984017 GGCCCATCCCGGGCACCGGCGGTCAGCCGCTccaaccgtttaaaccatcccggtAG 2983962
