# Supplementary material for: Comparative Whole-Genome Analysis of Clinical Isolates Reveals Characteristic Architecture of Mycobacterium tuberculosis Pangenome
Source: PLoS One. 2015 Apr 8;10(4):e0122979. doi: 10.1371/journal.pone.0122979 (PMC4390332; doi:10.1371/journal.pone.0122979)
Supplement: S1 Table — Detailed description of strains used in current study. (DOCX) [file pone.0122979.s009.docx]

**S1 Table Strains description. Detailed description of strains used in current study**

|  | MDR |
| --- | --- |
|  | XDR |

| **Organism** | **Size (Mb)** | **RefSeq** | **Scaffold** | **N's** | **Prodigal**  **predict** | **Status** | **Platform** | **Coverage** | **Assembly method** | **Type of Assembly** | **PMID** |
| --- | --- | --- | --- | --- | --- | --- | --- | --- | --- | --- | --- |
| Mafricanum | 4.39 | NC_015758 | 1 | no | 4041 | Complete | 454,Illumina GAII,ABI3730 | 10X | - | - | 22389744 |
| Mbov AF2122/97 | 4.35 | NC_002945 | 1 | no | 4009 | Scaffolds or contigs | Abi | - | PHRAP | - | 12788972 |
| Mbov AN5 | 4.33 | AWPL01 | 70 | no | 4051 | Scaffolds or contigs | - | - | - | - | Unpublished |
| Mbov BCG str. ATCC 35733 | 4.26 | AEZF01 | 32 | no | 4024 | Scaffolds or contigs | Illumina Genome Analyzer (Solexa) | 65.6x | SOAPdenovo v. 1.03 | Reference based denovo | 21478353 |
| Mbov BCG str. ATCC 35740 | 4.27 | AEZG01 | 36 | no | 4047 | Scaffolds or contigs | Illumina Genome Analyzer (Solexa) | 59.1x | SOAPdenovo v. 1.03 | Reference based denovo | 21478353 |
| Mbov BCG str. ATCC 35743 | 4.26 | AEZH01 | 28 | no | 4036 | Scaffolds or contigs | Illumina Genome Analyzer (Solexa) | 70.7x | SOAPdenovo v. 1.03 | Reference based denovo | 21478353 |
| Mbov BCG str. China | 4.26 | AEZE01 | 29 | no | 4039 | Scaffolds or contigs | Illumina Genome Analyzer (Solexa) | 63.2x | SOAPdenovo v. 1.03 | Reference based denovo | 21478353 |
| Mbov BCG str. Frappier | 4.24 | AKYQ01 | 178 | no | 4015 | Scaffolds or contigs | Illumina GAIIx | 100.0x | Velvet v. 1.0.12 | - | 23977002 |
| Mbov BCG str. Glaxo | 4.21 | AKYR01 | 327 | no | 4050 | Scaffolds or contigs | Illumina GAIIx | 100.0x | Newbler v. 1.0.12 | - | 23977002 |
| Mbov BCG str. Korea 1168P | 4.38 | NC_020245 | 1 | no | 4028 | Complete | - | - | - | - | Unpublished |
| Mbov BCG str. Moreau | 4.16 | AKYS01 | 540 | no | 4505 | Scaffolds or contigs | Illumina GAIIx | 100.0x | Velvet v. 1.0.12 | - | 23977002 |
| Mbov BCG str. Pasteur 1173P2 | 4.37 | NC_008769 | 1 | no | 4026 | Complete | ABI 3700 | - | PHRAP and GAP4 | - | 17372194 |
| Mbov BCG str. Phipps | 4.13 | AKYT01 | 810 | no | 4103 | Scaffolds or contigs | Illumina GAIIx | 100.0x | Velvet v. 1.0.12 | - | 23977002 |
| Mbov BCG str. Prague | 4.13 | AKYU01 | 896 | no | 4114 | Scaffolds or contigs | Illumina GAIIx | 100.0x | Velvet v. 1.0.12 | - | 23977002 |
| Mbov BCG str. Sweden | 4.08 | AKYV01 | 1092 | no | 4147 | Scaffolds or contigs | Illumina GAIIx | 100.0x | Velvet v. 1.0.12 | - | 23977002 |
| Mbov BCG str. Tokyo 172 | 4.37 | NC_012207 | 1 | no | 4034 | Complete | - | - | - | - | 19200449 |
| Mcan CIPT 140010059 | 4.48 | NC_015848 | 1 | no | 4070 | Complete | Sanger, 454/Roche, Illumina | - | Velvet | Reference based denovo | 23291586 |
| Mcan CIPT 140060008 | 4.43 | NC_019950 | 1 | yes | 4059 | Complete | Sanger, 454/Roche, Illumina | - | Velvet | Reference based denovo | 23291586 |
| Mcan CIPT 140070002 | 4.31 | CAOL01 | 587 | no | 4105 | Scaffolds or contigs | Sanger, 454/Roche, Illumina | - | Velvet | Reference based denovo | 23291586 |
| Mcan CIPT 140070005 | 4.30 | CAOM01 | 516 | no | 4058 | Scaffolds or contigs | Sanger, 454/Roche, Illumina | - | Velvet | Reference based denovo | 23291586 |
| Mcan CIPT 140070007 | 4.41 | CAOO01 | 502 | no | 4208 | Scaffolds or contigs | Sanger, 454/Roche, Illumina | - | Velvet | Reference based denovo | 23291586 |
| Mcan CIPT 140070008 | 4.42 | NC_019965 | 1 | yes | 4062 | Complete | Sanger, 454/Roche, Illumina | - | Velvet | Reference based denovo | 23291586 |
| Mcan CIPT 140070010 | 4.53 | NC_019951 | 1 | yes | 4124 | Complete | Sanger, 454/Roche, Illumina | - | Velvet | Reference based denovo | 23291586 |
| Mcan CIPT 140070013 | 4.34 | CAON01 | 542 | no | 4124 | Scaffolds or contigs | Sanger, 454/Roche, Illumina | - | Velvet | Reference based denovo | 23291586 |
| Mcan CIPT 140070017 | 4.52 | NC_019952 | 1 | yes | 4116 | Complete | Sanger, 454/Roche, Illumina | - | Velvet | Reference based denovo | 23291586 |
| Morygis | 4.28 | APKD01 | 108 | no | 4017 | Scaffolds or contigs | Illumina HiSeq | 60.0x | Velvet v. 1.2.03 | - | Unpublished |
| Mtb 02_1987 | 4.57 | ABLM01 | 22 | yes | 4352 | Scaffolds or contigs | ABI | 9X | - | - | Unpublished |
| Mtb 210 | 4.40 | ADAB01 | 130 | no | 4231 | Scaffolds or contigs | Sanger | 6.7x | - | - | Unpublished |
| Mtb 43-16836 | 4.38 | ATNF01 | 154 | no | 4149 | Scaffolds or contigs | 454; IonTorrent | 40.0x | MIRA v. 2013 | - | 24092792 |
| Mtb 7199-99 | 4.42 | NC_020089 | 1 | no | 4075 | Complete | 454 pyrosequencing | - | GS De Novo Assembler | Reference based denovo | 23424287 |
| Mtb 94_M4241A | 4.49 | ABLL01 | 10 | yes | 4302 | Scaffolds or contigs | ABI | 9X | - | - | Unpublished |
| Mtb '98-R604 INH-RIF-EM' | 4.34 | ABVM01 | 16 | yes | 4183 | Scaffolds or contigs | - | 6.9X | - | - | Unpublished |
| Mtb BTB05-552 | 4.36 | AEGC01 | 1 | no | 4109 | Scaffolds or contigs | 454 platform | 24x | CLC Genomics Workbench v. 3.7 | combinational | 21304944 |
| Mtb BTB05-559 | 4.34 | AEGD01 | 1 | no | 4081 | Scaffolds or contigs | 454 platform | 32x | CLC Genomics Workbench v. 3.7 | combinational | 21304944 |
| Mtb C | 4.38 | AAKR01 | 4 | yes | 4276 | Scaffolds or contigs | - | - | - | - | Unpublished |
| Mtb CCDC5079 | 4.40 | NC_017523 | 1 | no | 4190 | Complete | Solexa | 10x | Velvet | - | 21914894 |
| Mtb CCDC5079 | 4.41 | NC_021251 | 1 | no | 4088 | Complete | Roche 454 | - | Newbler Assembler | - | 23627759 |
| Mtb CCDC5180 | 4.41 | NC_017522 | 1 | no | 4105 | Complete | Solexa | 10x | Velvet | - | 21914894 |
| Mtb CDC1551 | 4.40 | NC_002755 | 1 | no | 4080 | Complete | - | - | - | - | 19099550 |
| Mtb CDC1551A | 4.38 | AELF01 | 53 | yes | 4138 | Scaffolds or contigs | 454 platform | 28.0x | Newbler v. 2.3 | - | Unpublished |
| Mtb CPHL_A | 4.42 | ACHP01 | 6 | yes | 4195 | Scaffolds or contigs | ABI | 9.15X | - | - | Unpublished |
| Mtb CTRI-2 | 4.40 | NC_017524 | 1 | no | 4073 | Complete | - | - | - | - | Unpublished |
| Mtb CTRI-4 | 4.32 | AIIE01 | 44 | no | 4048 | Scaffolds or contigs | Sanger dideoxy sequencing; 454 | 11x | GS De Novo Assembler v. 1.1.03.24 | - | 23437175 |
| Mtb EAI/OSDD271 | 4.17 | AQQC01 | 182 | no | 4248 | Scaffolds or contigs | 454 Flx; Ion Torrent PGM | 74.35x | CLC genomic workbench v. 6 | - | 23908284 |
| Mtb EAS054 | 4.43 | ABOV01 | 6 | yes | 4240 | Scaffolds or contigs | ABI | 9X | - | - | Unpublished |
| Mtb F11 | 4.42 | NC_009565 | 1 | no | 4085 | Complete | - | - | - | - | Unpublished |
| Mtb FJ05194 | 4.36 | ANBL01 | 112 | small N's | 4113 | Scaffolds or contigs | Illumina HiSeq | 110x | SOAPdenovo v. 1.05 | - | 23965132 |
| Mtb GM 1503 | 4.36 | ABQG01 | 17 | yes | 4367 | Scaffolds or contigs | ABI | 5.09X | - | - | Unpublished |
| Mtb GuangZ0019 | 4.37 | ANFI01 | 97 | yes | 4128 | Scaffolds or contigs | llumina HiSeq | 110x | SOAPdenovo v. 1.05 | - | 23965132 |
| Mtb H37Ra | 4.33 | AAYK01 | 272 | no | 4292 | Scaffolds or contigs | - | - | - | - | Unpublished |
| Mtb H37Ra | 4.42 | NC_009525 | 1 | no | 4095 | Complete | Shotgun sequencing | - | Arachne and Phrap | - | 18584054 |
| Mtb H37Rv | 4.41 | NC_000962 | 1 | no | 4086 | Complete | - | - | - | - | 9634230 |
| Mtb H37Rv | 4.41 | NC_018143 | 1 | no | 4077 | Complete | 454/Sanger/Illumina | - | Newbler v. 2.1 | - | Unpublished |
| Mtb H37RvCO | 4.41 | AJSF01 | 1 | small N's | 4088 | Scaffolds or contigs | Illumina GAII | 79.2x | Sacchettini lab sequence assembler v. | - | 20472797 |
| Mtb K85 | 4.43 | ACHQ01 | 1 | yes | 4159 | Scaffolds or contigs | ABI | 10.4X | - | - | Unpublished |
| Mtb KZN 1435 | 4.40 | NC_012943 | 1 | no | 4079 | Complete | - | - | - | - | Unpublished |
| Mtb KZN 4207 | 4.39 | NC_016768 | 1 | no | 4065 | Complete | - | - | - | - | Unpublished |
| Mtb KZN 605 | 4.40 | NC_018078 | 1 | no | 4079 | Complete | - | - | - | - | Unpublished |
| Mtb NA-A0008 | 4.38 | ALYG01 | 280 | no | 4375 | Scaffolds or contigs | IonTorrent | 89.0x | MIRA v. 2 | - | Unpublished |
| Mtb NA-A0009 | 4.39 | ALYH01 | 310 | no | 4404 | Scaffolds or contigs | IonTorrent | 93.0x | MIRA v. 2 | - | Unpublished |
| Mtb NCGM2209 | 4.52 | BADQ01 | 107 | yes | 4195 | Scaffolds or contigs | 454 GS FLX | 22x | GS De Novo Assembler v. 2.5.3 | - | 22072647 |
| Mtb OSDD 326 | 4.29 | SRR786668 | 450 | no | 4125 | Scaffolds or contigs | Illumina GA IIx | 465x | Velvet v. 1.1.08 | - | Unpublished |
| Mtb OSDD 386 | 4.23 | SRR784917 | 565 | no | 4229 | Scaffolds or contigs | Illumina GA IIx | 210x | Velvet v. 1.1.10 | - | Unpublished |
| Mtb OSDD 472 | 4.30 | SRR786667 | 432 | no | 4108 | Scaffolds or contigs | Illumina GA IIx | 640x | Velvet v. 1.1.07 | - | Unpublished |
| Mtb OSDD 487 | 4.30 | SRR786669 | 383 | no | 4173 | Scaffolds or contigs | Illumina GA IIx | 204x | Velvet v. 1.1.11 | - | Unpublished |
| Mtb OSDD 630 | 4.26 | SRR786188 | 411 | no | 4123 | Scaffolds or contigs | Illumina GA IIx | 253x | Velvet v. 1.1.09 | - | Unpublished |
| Mtb OSDD071 | 4.29 | SRR786373 | 393 | no | 4145 | Scaffolds or contigs | Illumina GA IIx | 390x | Velvet v. 1.1.06 | - | Unpublished |
| Mtb OSDD105 | 4.27 | AUXD02 | 181 | no | 4448 | Scaffolds or contigs | Ion-Torrent PGM | 395x | CLC Genomic Work Bench v. 6 NGS Cell | no | 24265496 |
| Mtb OSDD493 | 4.23 | AVQJ01 | 193 | no | 4303 | Scaffolds or contigs | IonTorrent | 402.0x | CLC NGS Cell v. 5.6 | no | 24201204 |
| Mtb OSDD504 | 4.23 | SRR786397 | 631 | no | 4202 | Scaffolds or contigs | Illumina GA IIx | 269x | Velvet v. 1.1.06 | - | Unpublished |
| Mtb OSDD518 | 4.27 | SRR786670 | 404 | no | 4132 | Scaffolds or contigs | Illumina GA IIx | 200x | Velvet v. 1.1.06 | - | Unpublished |
| Mtb PR05 | 4.32 | AOMG02 | 225 | no | 4142 | Scaffolds or contigs | Illumina GAIIx | 205.0x | CLCBio Genomic Workbench v. 5.5.1 | - | Unpublished |
| Mtb S96-129 | 4.34 | AEGB01 | 1 | no | 4112 | Scaffolds or contigs | 454 platform | 23x | CLC Genomics Workbench v. 3.7 | combinational | 21304944 |
| Mtb SP21 | 4.45 | AOUF01 | 30 | yes | 4364 | Scaffolds or contigs | 454; IonTorrent | 7.0x | GS De Novo Assembler v. 2.8; sspace v. basic 1.0 | - | Unpublished |
| Mtb str. Erdman = ATCC 35801 | 4.39 | NC_020559 | 1 | no | 4095 | Complete | GS FLX Titanium sequencer (Roche) | - | GS De Novo Assembler 2.6 (Newbler; Roche) | - | 22535945 |
| Mtb str. Haarlem | 4.41 | NC_022350 | 1 | no | 4063 | Complete | - | - | - | - | Unpublished |
| Mtb str. OSDD515 | 4.21 | AUXC01 | 127 | no | 4197 | Scaffolds or contigs | IonTorrent PGM | 386x | CLC NGS Cell v. 5.6 | no | 24265488 |
| Mtb SUMu001 | 4.38 | ADHQ01 | 79 | yes | 4188 | Scaffolds or contigs | 454 platform | 19.5x | - | - | Unpublished |
| Mtb SUMu002 | 4.40 | ADHR01 | 40 | yes | 4246 | Scaffolds or contigs | 454 platform | 18.1x | - | - | Unpublished |
| Mtb SUMu003 | 4.38 | ADHS01 | 63 | yes | 4175 | Scaffolds or contigs | 454 platform | 30.1x | - | - | Unpublished |
| Mtb SUMu004 | 4.37 | ADHT01 | 64 | yes | 4178 | Scaffolds or contigs | 454 platform | 28.94x | - | - | Unpublished |
| Mtb SUMu005 | 4.38 | ADHU01 | 55 | yes | 4170 | Scaffolds or contigs | 454 platform | 18.82x | - | - | Unpublished |
| Mtb SUMu006 | 4.37 | ADHV01 | 58 | yes | 4193 | Scaffolds or contigs | 454 platform | 16.37x | - | - | Unpublished |
| Mtb SUMu007 | 4.36 | ADHW01 | 41 | yes | 4160 | Scaffolds or contigs | 454 platform | 22.39x | - | - | Unpublished |
| Mtb SUMu008 | 4.38 | ADHX01 | 41 | yes | 4183 | Scaffolds or contigs | 454 platform | 26.17x | - | - | Unpublished |
| Mtb SUMu009 | 4.38 | ADHY01 | 46 | yes | 4153 | Scaffolds or contigs | 454 platform | 18.18x | - | - | Unpublished |
| Mtb SUMu010 | 4.41 | ADHZ01 | 52 | yes | 4191 | Scaffolds or contigs | 454 platform | 23.16x | - | - | Unpublished |
| Mtb SUMu011 | 4.42 | ADIA01 | 56 | yes | 4248 | Scaffolds or contigs | 454 platform | 15.77x | - | - | Unpublished |
| Mtb SUMu012 | 4.40 | ADIB01 | 93 | yes | 4306 | Scaffolds or contigs | 454 platform | 15.89x | - | - | Unpublished |
| Mtb T17 | 4.43 | ABQH01 | 22 | yes | 4543 | Scaffolds or contigs | ABI | 4.26X | - | - | Unpublished |
| Mtb T46 | 4.41 | ACHO01 | 8 | yes | 4302 | Scaffolds or contigs | ABI | 5.9X | - | - | Unpublished |
| Mtb T85 | 4.42 | ABOW01 | 17 | yes | 4421 | Scaffolds or contigs | ABI | 5X | - | - | Unpublished |
| Mtb T92 | 4.38 | ABLN01 | 31 | yes | 4524 | Scaffolds or contigs | ABI | 4.5X | - | - | Unpublished |
| Mtb UM 1072388579 | 4.28 | AMXW01 | 89 | yes | 4035 | Scaffolds or contigs | Illumina HiSeq | 100.0x | Velvet v. 1.1.07 | - | 23405310 |
| Mtb UT205 | 4.42 | NC_016934 | 1 | yes | 4120 | Complete | ROCHE 454-GS-FLX TITANIUM | 20x | newbler v2.3 | - | 22404577 |
| Mtb W-148 | 4.54 | ACSX01 | 1 | yes | 4270 | Scaffolds or contigs | Sanger | 10.8x | - | - | Unpublished |
