# Supplementary material for: Comparative Whole-Genome Analysis of Clinical Isolates Reveals Characteristic Architecture of Mycobacterium tuberculosis Pangenome
Source: PLoS One. 2015 Apr 8;10(4):e0122979. doi: 10.1371/journal.pone.0122979 (PMC4390332; doi:10.1371/journal.pone.0122979)
Supplement: S2 Table — List of forward and reverse primers used for the validation and their expected product sizes. (DOCX) [file pone.0122979.s010.docx]

**S2 Table Primers used.** List of forward and reverse primers used for the validation and their expected product sizes.

| **Name** | **Forward Primer** | **Reverse primer** | **Expected product size(bp)** | **Annealing temperature used (˚C)** |
| --- | --- | --- | --- | --- |
| Gene-1 | GTGCGGCCTTGGTGCTGACC | CGCGCGCAACTTCTGCACTG | 728 | 64 |
| Gene-2 | CACGGTCGGGTTCACAGGGC | CGCGGAATCGCTACCGCCAT | 561 | 64 |
| Gene-3 | GGGGCGGATTCGCACAGCAT | CAGCCTGGACGACGACTCGC | 547 | 64 |
| Gene-4 | GCAACAGAATGGCACGCGCA | CGACAGCGATCTGACGTTGCGT | 496 | 62 |
| Gene-5 | CCCGACATCGCCACCGTCTT | CACCATCTGCGCTTTCGGTGC | 266 | 64 |
| Gene-6 | AGTCCCTGAGGTCGGCAATCGT | CATCGCTGGCGTTCGTTGAACC | 225 | 68 |
| Gene-7 | CGCATGACGGTCCAAGACGTG | GGCGCTAGCTCGCCCTTGTC | 224 | 66 |
| Gene-8 | GTGTGCAGCGCGCGATTGAG | ACCGGGATGGTTTAAACGGTTGG | 195 | 64 |
| Gene-9 | ACCGCCGCTTTCTCCTTCGC | AAGCGCGCACTTCTCGGGG | 147 | 62 |
